# Supplementary material for: Development and Validation of a Parameter-Free Model Chemistry for the Computation of Reliable Reaction Rates
Source: J Chem Theory Comput. 2021 Jul 6;17(8):4913–28. doi: 10.1021/acs.jctc.1c00406 (PMC8359010; doi:10.1021/acs.jctc.1c00406)
Supplement: Supplementary file 1 — ct1c00406_si_001.pdf [file ct1c00406_si_001.pdf]

Supporting Information

Development and validation of a parameter-free model chemistry for the computation of reliable reaction rates

Vincenzo Barone,* Jacopo Lupi, Zoi Salta, and Nicola Tasinato

SMART Laboratory, Scuola Normale Superiore di Pisa, piazza dei Cavalieri 7, 56125 Pisa, Italy

E-mail: vincenzo.barone@sns.it

Extended Table of results for the HTBH38/08 dataset

Table 1: Theoretical values of the barrier heights for the forward and reverse reactions in the HTBH38/08 and NHTBH38/08 datasets, obtained at different levels of theory. All values (not including zero-point energy and spin-orbit corrections) are in kcal mol^{-1}.

	Reaction	forward/reverse barrier height					
		revDSD[a]	CCSD(T)/revDSD[b]	CCSD(T)/QCISD[c]	jChS/revDSD[d]	jChS/QCISD[e]	"best"[f]
c1	$H^\bullet + HCl \longrightarrow H_2 + Cl^\bullet$	1.27/7.54	5.63/9.07	5.90/9.34	4.97/7.80	5.57/7.95	5.49/7.42[g]
c2	$OH^\bullet + H_2 \longrightarrow H_2O + H^\bullet$	5.88/20.92	6.04/20.92	5.97/20.84	5.47/21.76	5.38/21.69	5.10/21.20
c3	$CH_3{}^\bullet + H_2 \longrightarrow CH_4 + H^\bullet$	11.32/14.36	12.30/14.99	12.29/14.97	11.96/14.64	11.95/14.64	12.10/15.30
c4	$H^\bullet + H_2 \longrightarrow H_2 + H^\bullet$	8.96/8.96	10.02/10.02	10.01/10.01	9.58/9.58	9.58/9.58	9.60/9.60
c5	$OH^\bullet + NH_3 \longrightarrow H_2O + NH_2{}^\bullet$	4.67/14.37	4.21/14.29	4.18/14.27	3.93/14.41	3.35/13.85	3.20/12.70
c6	$HCl + CH_3{}^\bullet \longrightarrow Cl^\bullet + CH_4$	1.09/10.40	2.27/8.39	2.27/8.39	1.69/6.35	1.70/6.77	1.70/7.90
c7	$OH^\bullet + C_2H_6 \longrightarrow H_2O + C_2H_5{}^\bullet$	4.47/19.97	4.17/19.71	4.03/19.57	3.80/20.91	3.64/20.75	3.40/19.90
c8	$F^\bullet + H_2 \longrightarrow HF + H^\bullet$	2.77/32.22	2.26/32.76	2.26/32.75	1.69/33.90	1.77/34.00	1.80/33.40
c9	$^3O + CH_4 \longrightarrow OH^\bullet + CH_3{}^\bullet$	14.60/8.24	15.33/8.42	15.36/8.45	14.55/9.63	14.65/9.62	13.70/8.10
c10	$H^\bullet + PH_3 \longrightarrow PH_2{}^\bullet + H_2$	2.61/24.50	3.33/25.90	3.30/25.88	2.85/25.09	2.82/25.05	3.10/23.20
c11	$O + HCl \longrightarrow OH^\bullet + Cl^\bullet$	7.60/10.56	11.67/10.89	11.61/10.81	10.59/10.34	10.63/10.66	9.80/10.40
c12	$NH_2{}^\bullet + CH_3{}^\bullet \longrightarrow CH_4 + NH$	8.94/21.97	8.69/22.99	8.69/23.01	9.49/22.09	9.50/22.11	8.00/22.40
c13	$NH_2{}^\bullet + C_2H_5 \longrightarrow NH + C_2H_5$	9.28/18.81	9.01/19.98	9.43/20.40	9.97/19.08	10.39/19.51	7.50/18.30
c14	$NH_2{}^\bullet + C_2H_6 \longrightarrow NH_3 + C_2H_5{}^\bullet$	11.62/17.43	11.85/17.31	11.83/17.28	11.24/17.85	11.18/17.80	10.40/17.40
c15	$NH_2{}^\bullet + CH_4 \longrightarrow NH_3 + CH_3{}^\bullet$	14.11/46.42	14.47/16.59	14.47/16.58	13.82/16.94	13.80/16.92	14.50/17.80
c16	s-trans cis$-C_5H_8 \longrightarrow$ same	39.42/39.42	39.87/39.87	39.87/39.87	39.66/39.66	39.63/39.63	38.40/38.40
c17	$H^\bullet + FH \longrightarrow HF + H^\bullet$	40.68/40.68	42.64/42.64	42.64/42.64	41.99/41.99	42.02/42.02	42.18/42.18
c18	$H^\bullet + FCH_3 \longrightarrow HF + CH_3{}^\bullet$	30.97/55.29	30.51/57.43	30.47/57.40	30.31/57.54	30.31/57.54	30.38/57.02
c19	$H^\bullet + F_2 \longrightarrow HF + F^\bullet$	9.32/111.60	5.07/108.93	1.85/105.78	2.38/106.07[h]	1.49/105.25	2.59/105.77[i]
c20	$F^- + CH_3F \longrightarrow FCH_3 + F^-$	-0.71/-0.71	-0.02/-0.02	-0.04/-0.04	-0.70/-0.70	-0.71/-0.71	-0.34/-0.34
c21	$F^-\cdots CH_3F \longrightarrow FCH_3\cdots F^-$	12.72/12.72	13.53/13.53	13.50/13.50	13.21/13.21	13.20/13.20	13.38/13.38
c22	$Cl^- + CH_3Cl \longrightarrow ClCH_3 + Cl^-$	1.86/1.86	3.11/3.11	3.12/3.12	2.27/2.27	2.33/2.33	3.10/3.10
c23	$F^- + CH_3Cl \longrightarrow FCH_3 + Cl^-$	-12.48/19.02	-11.73/20.09	-11.73/20.08	-12.32/19.29	-12.31/19.31	-12.54/20.11
c24	$OH^-\cdots CH_3F \longrightarrow HOCH_3\cdots F^-$	10.42/47.21	11.18/47.80	11.15/47.76	11.14/47.38	11.14/47.38	10.96/47.20
c25	$H^\bullet + CO \longrightarrow HCO^\bullet$	3.52/23.65	3.74/22.52	3.71/22.50	3.22/22.87	3.19/22.82	3.17/22.68
c26	$CH_3{}^\bullet + C_2H_4 \longrightarrow CH_3CH_2CH_2{}^\bullet$	7.91/35.57	6.74/32.64	6.61/32.51	6.37/32.77	6.35/32.74	6.85/32.97
	MUE	1.27/2.18 (0.89/2.25)	0.73/0.71 (0.61/0.60)	0.64/0.63 (0.61/0.63)	0.70/0.88 (0.50/0.58)	0.52/0.66 (0.50/0.59)	-
	RMSD	1.91/5.84 (1.02/6.49)	0.99/1.05 (0.82/0.89)	0.85/0.90 (0.84/0.93)	1.04/1.23 (0.74/0.73)	0.80/0.80 (0.81/0.74)	-

[a]revDSD-PBEP86-D3(BJ)/jun-cc-pV(T+*d*)Z;[b]CCSD(T)/ jun-cc-pV(T+*d*)Z//revDSD-PBEP86-D3(BJ)/jun-cc-pV(T+d)Z;[c]CCSD(T)/ jun-cc-pV(T+d)Z//QCISD/MG3;[d]jChS on revDSD-PBEP86-D3(BJ)/jun-cc-pV(T+d)Z optimized geometry;[e]jChS on QCISD/MG3 optimized geometry;[f] Zheng *et al. J. Chem. Theory Comput.* 2009, *5*, 808–821; [g] CBS-CVH result (this work): the original values were 5.49/7.42; [h] jChS geometry; the values at the rev-DSD geometry are: 4.46/108.14; [i] CBS-CVH result (this work): the original values were 2.27/105.80

XYZ geometries

rev-DSD geometries from Table 1

N_2O

3

N	0.00000000	0.00000000	-1.20449800
N	0.00000000	0.00000000	-0.06931700
O	0.00000000	0.00000000	1.11458800

OH

2

O	0.00000000	0.00000000	0.10796800
H	0.00000000	0.00000000	-0.86374100

N_2

2

N	0.00000000	0.00000000	0.55106900
N	0.00000000	0.00000000	-0.55106900

HCl

2

Cl	0.00000000	0.00000000	0.07091900
H	0.00000000	0.00000000	-1.20561900

CH_3

4

C	0.00000000	0.00000000	0.00000100
H	0.00000000	0.00000000	1.07836500
H	0.00000000	0.93388900	-0.53918400
H	0.00000000	-0.93388900	-0.53918400

FCl

2

F	0.00000000	0.00000000	-1.06890300
Cl	0.00000000	0.00000000	0.56589000

CH_3F

5

C	-0.11963200	0.23669900	0.57744300
F	0.14156200	-0.28008900	-0.68329500
H	-0.41636400	-0.58026000	1.23539400
H	0.78523800	0.71418400	0.95372800
H	-0.92513700	0.96668400	0.49587700

$Cl^{-}\cdots CH_3Cl$

6

Cl	0.00021800	2.37466100	0.00000000

C	0.00000000	0.55243500	0.00000000
H	1.02753300	0.21197500	0.00000000
H	-0.51382900	0.21215200	0.88990200
H	-0.51382900	0.21215200	-0.88990200
Cl	-0.00021000	-2.60706600	0.00000000

$F^{-}\cdots CH_3Cl$

6

Cl	0.00000000	0.00000000	1.62730400
C	0.00000000	0.00000000	-0.22587400
H	0.00000000	1.02932800	-0.55203400
H	0.89142400	-0.51466400	-0.55203400
H	-0.89142400	-0.51466400	-0.55203400
F	0.00000000	0.00000000	-2.73920200

$FCH_3\cdots Cl^{-}$

6

F	0.00011000	2.64364200	0.00000000
C	0.00000000	1.22601700	0.00000000
H	1.02766800	0.87517100	0.00000000
H	-0.51387800	0.87528800	0.89000800
H	-0.51387800	0.87528800	-0.89000800
Cl	-0.00005300	-1.98674300	0.00000000

OH^{-}

2

O	0.00000000	0.00000000	0.10741400
H	0.00000000	0.00000000	-0.85931500

$HOCH_3$

6

C	-0.04671600	0.66506200	0.00000000
O	-0.04671600	-0.75718100	0.00000000
H	-1.08963800	0.97778100	0.00000000
H	0.86530900	-1.05817500	0.00000000
H	0.43917800	1.07373600	0.89160500
H	0.43917800	1.07373600	-0.89160500

HN_2

3

N	-0.06194600	0.65702500	0.00000000
N	-0.06194600	-0.51361200	0.00000000
H	0.86725100	-1.00388900	0.00000000

C_2H_4

6

C	0.00000000	0.00000000	0.66586300
C	0.00000000	0.00000000	-0.66586300

```
H                       0.00000000    0.92390200    1.23192700
H                       0.00000000   -0.92390200    1.23192700
H                       0.00000000    0.92390200   -1.23192700
H                       0.00000000   -0.92390200   -1.23192700
```

C_2H_5

```
7


C                       0.79370900    0.00000000   -0.02223600
C                      -0.69434100    0.00000000   -0.00075100
H                      -1.10385100    0.88509600   -0.49249500
H                       1.34818600   -0.92501100    0.04802900
H                      -1.08488000    0.00000400    1.02685000
H                      -1.10385100   -0.88510000   -0.49248700
H                       1.34818600    0.92501100    0.04802900
```

HCN

```
3


C                       0.00000000    0.00000000   -0.50248700
N                       0.00000000    0.00000000    0.65497200
H                       0.00000000    0.00000000   -1.56988000
```

HNC

```
3


C                       0.00000000    0.00000000   -0.74075100
```

N	0.00000000	0.00000000	0.43090100
H	0.00000000	0.00000000	1.42820500

CH_4

5

H	0.00000000	0.00000000	1.08935100
C	0.00000000	0.00000000	0.00000100
H	0.00000000	1.02704900	-0.36311900
H	0.88945000	-0.51352400	-0.36311900
H	-0.88945000	-0.51352400	-0.36311900

H_2O

3

H	0.00000000	0.75969600	-0.47089100
O	0.00000000	0.00000000	0.11772300
H	0.00000000	-0.75969600	-0.47089100

H_2

2

H	0.00000000	0.00000000	0.37111100
H	0.00000000	0.00000000	-0.37111100

H_2S

3

S	0.00000000	0.00000000	0.10283900
H	0.00000000	0.96667600	-0.82271600
H	0.00000000	-0.96667600	-0.82271600

HS

2

S	0.00000000	0.00000000	0.07895200
H	0.00000000	0.00000000	-1.26323400

TS a1

4

H	-0.28672400	-1.90580400	0.00000000
O	-0.84331200	-0.64877200	0.00000000
N	0.00000000	0.25005100	0.00000000
N	1.00474500	0.76366000	0.00000000

TS a2

3

H	0.00000000	0.00000000	1.47241200
Cl	0.00000000	0.00000000	0.00000000
H	0.00000000	0.00000000	-1.47241200

TS a3

6

Cl	-0.01883600	-0.16267900	-1.42378200
F	0.00423300	0.03653300	0.31971100
C	0.03087500	0.26668400	2.33406700
H	0.46724300	1.25305400	2.32376700
H	-1.03483100	0.16734500	2.46768800
H	0.66444700	-0.58375300	2.53103100

TS a4

6

Cl	0.00223800	0.07207200	2.31171800
C	0.00004200	0.00000200	0.00000000
H	1.07057500	0.03261400	-0.00205800
H	-0.50693500	-0.94296300	0.02989200
H	-0.56350400	0.91035700	-0.02783400
Cl	-0.00226100	-0.07207300	-2.31171800

TS a5

6

F	-0.00000600	2.53726300	0.00000000
C	0.00000000	0.48884500	0.00000000
H	1.06442400	0.61446100	0.00000000
H	-0.53221200	0.61445500	0.92181900
H	-0.53221200	0.61445500	-0.92181900

Cl	0.00000300	-1.62422400	0.00000000

TS a6

7

F	1.85894200	-0.01120900	0.00000000
C	0.09868900	0.00683800	0.00000000
H	0.03897700	1.07773800	-0.00000200
H	0.03933100	-0.52279000	-0.93079900
H	0.03933100	-0.52278700	0.93080100
O	-1.90573700	0.10540100	0.00000000
H	-2.19435100	-0.81551500	0.00000000

TS a7

3

N	0.08169700	-0.64381200	0.00000000
N	0.08169700	0.47395400	0.00000000
H	-1.14375200	1.18900300	0.00000000

TS a8

7

C	0.56786200	0.00000000	-0.21084400
C	-0.74556700	0.00000000	0.03764800
H	1.44928100	-0.00000200	1.50772900
H	1.10461300	-0.92212500	-0.39688400

H	1.10461200	0.92212600	-0.39688200
H	-1.29613900	0.92392700	0.16260900
H	-1.29613900	-0.92392700	0.16260700

TS a9

3

C	0.08061200	0.61925400	0.00000000
N	0.08061200	-0.56884400	0.00000000
H	-1.04796200	0.26638400	0.00000000

TS a10

7

C	1.21493800	-0.00887100	0.00002100
O	-1.30325700	0.10924900	0.00000400
H	0.01803300	0.12353700	0.00018100
H	1.47468900	-0.53859200	0.91255000
H	1.47250600	-0.56262700	-0.89876100
H	1.59400300	1.01016400	-0.01409000
H	-1.42280200	-0.85324600	-0.00003900

TS a11

3

H	0.66994500	-0.41203400	0.00000000
O	0.00000000	0.28747500	0.00000000
H	-0.66994500	-1.88776200	0.00000000

TS a12

4

H	1.26507000	-0.21794900	0.00000000
S	0.00000000	0.22302700	0.00000000
H	-0.49484900	-1.11057200	0.00000000
H	-0.77022100	-2.23991800	0.00000000

rev-DSD geometries from Table 2

HF

2

F	0.00000000	0.00000000	0.09210800
H	0.00000000	0.00000000	-0.82896800

F_2

2

F	0.00000000	0.00000000	0.70105300
F	0.00000000	0.00000000	-0.70105300

$F^{-}\cdots CH_3F$

6

F	-0.00025200	1.85774300	0.00000000
C	0.00000000	0.42346500	0.00000000

H	1.02703500	0.07809000	0.00000000
H	-0.51342300	0.07780600	0.88938100
H	-0.51342300	0.07780600	-0.88938100
F	0.00023100	-2.16602000	0.00000000

CH_3Cl

5

C	0.00000000	-1.12712700	0.00000000
Cl	0.00000000	0.65728000	0.00000000
H	1.03070500	-1.47033300	0.00000000
H	-0.51535200	-1.47033500	0.89261600
H	-0.51535200	-1.47033500	-0.89261600

$OH^{-}\cdots CH_3F$

7

F	-1.91031200	-0.06570700	0.00000400
C	-0.49045200	0.09360600	-0.00000500
H	-0.07922800	-0.37683200	0.88573100
H	-0.07923300	-0.37693100	-0.88569100
H	-0.27063400	1.15670800	-0.00006500
O	2.18342400	-0.07592400	-0.00000300
H	3.09722800	0.23417100	0.00004500

$HOCH_3\cdots F^{-}$

7

C	-1.30881600	-0.38528200	0.00000000
O	-0.47349300	0.72499800	0.00000000
H	-2.36139800	-0.06245400	-0.00001500
H	-1.16521500	-1.03114400	-0.88476000
H	-1.16523600	-1.03112900	0.88477400
H	0.52803100	0.35447000	0.00000100
F	1.75607300	-0.19089300	0.00000000

CO

2

O	0.00000000	0.00000000	0.48547300
C	0.00000000	0.00000000	-0.64729700

HCO

3

H	-0.85895000	1.22445300	0.00000000
C	0.06135400	0.58622500	0.00000000
O	0.06135400	-0.59272500	0.00000000

$CH_3CH_2CH_2$

10

C	1.20801100	-0.28741000	0.00006100
C	-0.06699700	0.57684200	-0.00006100

C	-1.31352300	-0.23961300	-0.00001100
H	1.24057100	-0.92923200	0.88298600
H	1.24059800	-0.92943600	-0.88271600
H	2.10233700	0.34062600	0.00000000
H	-0.04842000	1.22813100	-0.87902300
H	-0.04847400	1.22832900	0.87875100
H	-1.72595200	-0.61836100	0.92552200
H	-1.72560200	-0.61896800	-0.92545300

TS NHT1

3

H	0.00000000	0.00000000	1.13917200
F	0.00000000	0.00000000	0.00000000
H	0.00000000	0.00000000	-1.13917200

TS NHT2

6

H	-0.58871400	-0.83945800	1.70578700
F	-0.22493300	-0.32073100	0.65173200
C	0.26709400	0.38085000	-0.77389300
H	-0.52726100	1.08632400	-0.97600700
H	1.21871800	0.81869900	-0.50511600
H	0.31908900	-0.46408500	-1.44689100

TS NHT3

3

H	0.00000000	0.00000000	-2.15146300
F	0.00000000	0.00000000	-0.58150700
F	0.00000000	0.00000000	0.82055800CH3CH2CH2

TS NHT4

6

F	0.00146400	0.05174800	1.83052400
C	0.00000000	0.00000300	0.00000000
H	0.01227600	-1.07142600	0.03027900
H	-0.93439200	0.52511100	-0.01409800
H	0.92211700	0.54632400	-0.01618200
F	-0.00146400	-0.05175000	-1.83052400

TS NHT5 same as TS NHT4

TS NHT6 same as TS a4

TS NHT7 same as TS a5

TS NHT8 same as TS a6

TS NHT9

3

H	-1.55294800	1.39709400	0.00000000

C	0.11092500	0.54987900	0.00000000
O	0.11092500	-0.58704600	0.00000000

TS NHT10

10

C	-0.47567100	0.64170500	0.00000000
C	-1.37852700	-0.36149500	0.00000000
H	-0.22646300	1.15659100	-0.91922400
H	-0.22646300	1.15659100	0.91922400
H	-1.72825800	-0.80471700	0.92392800
H	-1.72825800	-0.80471700	-0.92392800
C	1.61125700	-0.24084300	0.00000000
H	2.18972000	0.67457000	-0.00000100
H	1.58868000	-0.80726300	-0.92055800
H	1.58868100	-0.80726100	0.92055900

rev-DSD geometries from Table 3

NH_3

4

N	0.00000000	0.00000000	0.11448400
H	0.00000000	0.93911400	-0.26712900
H	0.81329700	-0.46955700	-0.26712900
H	-0.81329700	-0.46955700	-0.26712900

NH_2

3

N	0.00000000	0.00000000	0.14168900
H	0.00000000	0.80311100	-0.49591000
H	0.00000000	-0.80311100	-0.49591000

C_2H_6

8

C	-0.04827700	-0.18287700	-0.73954500
C	0.04827700	0.18287700	0.73954500
H	1.08142200	0.14022000	1.09087900
H	0.32210000	-1.19391600	-0.92193700
H	-0.53946000	-0.50125800	1.35531200
H	-0.32209500	1.19391700	0.92193600
H	-1.08142300	-0.14022600	-1.09087700
H	0.53945600	0.50126200	-1.35531200

PH_3

4

P	0.00000000	0.00000000	0.12703000
H	0.00000000	1.19265400	-0.63515200
H	1.03286900	-0.59632700	-0.63515200
H	-1.03286900	-0.59632700	-0.63515200

PH_2

3

P	0.00000000	0.00000000	0.11591300
H	0.00000000	1.02116400	-0.86935100
H	0.00000000	-1.02116400	-0.86935100

NH

2

N	0.00000000	0.00000000	0.12948000
H	0.00000000	0.00000000	-0.90636200

TS HT1

3

H	0.00000000	0.00000000	-2.21052500
H	0.00000000	0.00000000	-1.22601200
Cl	0.00000000	0.00000000	0.20214900

TS HT2

4

O	0.30472300	-0.10771900	-0.00001100
H	0.42755600	0.85550300	0.00003000
H	-1.04029200	-0.11694900	0.00001500
H	-1.82504600	0.12319700	0.00004100

TS HT3

6

C	0.00000000	0.26867500	0.00000000
H	1.05620400	0.51471000	0.00000000
H	-0.52810300	0.51470700	0.91470000
H	-0.52810300	0.51470700	-0.91470000
H	0.00000100	-1.13209900	0.00000000
H	0.00000100	-2.02407300	0.00000000

TS HT4

3

H	0.00000000	0.00000000	0.00000000
H	0.00000000	0.00000000	0.92695800
H	0.00000000	0.00000000	-0.92695800

TS HT5

6

N	-1.15209900	-0.05370300	-0.09360700
O	1.18186400	-0.08933100	-0.01417400
H	-1.26785000	-0.46768800	0.82852200
H	-1.31832100	0.94522700	0.00824800
H	-0.08940900	-0.20128300	-0.38389300
H	1.28535900	0.81431200	0.31576300

TS HT6

6

C	0.27345300	0.53814400	1.71911200
H	1.18480100	1.11418200	1.61229600
H	0.36219700	-0.41711300	2.22249500
H	-0.63382700	1.10001000	1.90601700
H	0.06285700	0.12370200	0.39516800
Cl	-0.15392600	-0.30292000	-0.96768500

TS HT7

10

C	1.47328000	-0.44609800	0.02563600
C	0.47467600	0.69211600	-0.02785600
O	-1.87944100	-0.31389300	-0.05246800
H	1.31244600	-1.06992600	0.90604800
H	1.39457600	-1.08058000	-0.85839200
H	2.49526300	-0.05792800	0.07022600
H	0.47764800	1.31793300	0.86494500
H	0.55504300	1.30827400	-0.92361200
H	-0.60420500	0.21557900	-0.08961400
H	-2.28297800	0.40169000	0.46346300

TS HT8

3

H	0.25006300	-1.14652900	0.00000000
F	0.00000000	0.32045700	0.00000000
H	-0.25006300	-1.73758100	0.00000000

TS HT9

6

C	0.00070000	0.02702100	-1.04395900
H	0.02709100	1.09726200	-1.20666900
H	-0.93520300	-0.47795800	-1.24809300
H	0.91059400	-0.52346500	-1.24803300
H	-0.00011900	-0.00460100	0.17787000
O	-0.00082000	-0.03167000	1.22358500

TS HT10

5

P	0.21701600	0.00000000	-0.11225000
H	0.26709200	1.03090500	0.85756000
H	0.26710300	-1.03090000	0.85756600
H	-1.25861200	-0.00000600	-0.13528400
H	-2.53082800	0.00000000	0.10390800

TS HT11

3

Cl	0.01825000	-0.81940600	0.00000000

H	-0.45624300	0.54463300	0.00000000
O	0.01825000	1.67315800	0.00000000

TS HT12

7

C	-1.19384500	-0.01136400	0.00000000
N	1.39551200	0.13004100	0.00000000
H	-1.41734100	-0.55411600	0.91283500
H	-1.41733800	-0.55424000	-0.91276200
H	-1.51925300	1.02403300	-0.00007100
H	0.18284400	0.13055300	-0.00000200
H	1.56556800	-0.88833300	0.00000000

TS HT13

10

C	-1.39822200	-0.44485300	0.00000700
C	-0.42981900	0.71044700	-0.00000700
N	1.92687300	-0.37397900	-0.00001200
H	-1.26525300	-1.07213000	-0.88327800
H	-1.26525600	-1.07210700	0.88330800
H	-2.43389900	-0.08725300	0.00000000
H	-0.42060700	1.31739800	-0.90360100
H	-0.42060400	1.31741800	0.90357300
H	0.79556000	0.12926300	-0.00001100
H	2.49019000	0.49170600	0.00009600

TS HT14

11

C	-1.48924100	-0.44549000	-0.00002400
C	-0.50065600	0.69814200	0.00003400
N	1.86779000	-0.34239400	-0.00005300
H	-1.36205900	-1.07527800	-0.88238900
H	-1.36204600	-1.07538100	0.88226600
H	-2.51959400	-0.07454500	0.00000700
H	-0.51794200	1.31445400	-0.89830500
H	-0.51792100	1.31434700	0.89844600
H	0.65349300	0.14703500	-0.00003100
H	2.24555100	0.16537200	-0.80475500
H	2.24536700	0.16484100	0.80507100

TS HT15

8

C	-1.25631100	0.00000000	0.01233800
N	1.31177600	0.00000000	-0.13801200
H	-1.58551200	0.90751000	-0.48687700
H	-1.45412000	-0.00006900	1.08014300
H	-1.58552500	-0.90744000	-0.48699700
H	0.03417400	-0.00000100	-0.15929300
H	1.47320600	0.80546000	0.47253900
H	1.47320700	-0.80545900	0.47254000

TS HT16

13

C	-1.30158100	-0.90607500	-0.02048200
C	-1.20775300	0.50665300	-0.01259500
C	0.00000000	1.18410000	0.15328400
C	1.20775300	0.50665300	-0.01259500
C	1.30158100	-0.90607400	-0.02048200
H	2.17093600	-1.32925400	-0.52016100
H	1.03576900	-1.45554500	0.87644200
H	2.03901900	1.08789900	-0.40199500
H	-0.00000100	2.26603000	0.08622200
H	-2.03901900	1.08789700	-0.40199500
H	-2.17093400	-1.32925600	-0.52016100
H	0.00000000	-1.18376800	-0.51757100
H	-1.03576700	-1.45554500	0.87644200

jChS geometries from Table 4

HCl

2

H	0.00000000	0.00000000	0.00000000
Cl	0.00000000	0.00000000	1.27415090

H_2

2

H	0.00000000	0.00000000	0.00000000
H	0.00000000	0.00000000	0.74097310

OH

2

O	0.00000000	0.00000000	0.00000000
H	0.00000000	0.00000000	0.96809910

NH_3

4

N	-0.00000322	0.11355682	-0.00000557
H	-0.46818172	-0.26495985	0.81102387
H	-0.46825389	-0.26497806	-0.81098485
H	0.93645813	-0.26495985	-0.00000000

H_2O

3

O	0.00000000	0.00000000	-0.11712295
H	-0.75600683	0.00000000	0.46849179
H	0.75600683	0.00000000	0.46849179

NH_2

3

N	0.00000000	0.00000000	-0.14126973
H	-0.80120657	0.00000000	0.49444406
H	0.80120657	0.00000000	0.49444406

CH_4

5

C	0.00000000	0.00000000	0.00000000
H	-0.00000000	-0.00000000	1.08556369
H	-0.00000000	-1.02347927	-0.36185456
H	-0.88635904	0.51173963	-0.36185456
H	0.88635904	0.51173963	-0.36185456

CH_3

4

C	0.00000000	0.00000000	-0.00000000
H	0.00000000	1.07554100	0.00000000
H	0.93144583	-0.53777050	0.00000000
H	-0.93144583	-0.53777050	0.00000000

PH_3

4

P	0.00000000	0.00000000	-0.12746980
H	0.00000000	1.18641086	0.63734902

H	-1.02746195	-0.59320543	0.63734902
H	1.02746195	-0.59320543	0.63734902

PH_2

3

P	0.00000000	-0.00000000	-0.11573335
H	-1.01728832	0.00000000	0.86800012
H	1.01728832	0.00000000	0.86800012

NH

2

N	0.00000000	0.00000000	0.00000000
H	0.00000000	0.00000000	1.03484400

TS HT1

3

H	0.00000000	0.00000000	-2.21125700
H	0.00000000	0.00000000	-1.22280300
Cl	0.00000000	0.00000000	0.20200400

TS HT5

6

N	-1.14133900	-0.05189400	-0.09534500

H	-1.26271400	-0.48497400	0.81430000
H	-1.30792100	0.94205900	0.02696700
H	-0.05845900	-0.19831900	-0.38949500
O	1.17013500	-0.08908700	-0.01122900
H	1.25738400	0.81718500	0.30547300

TS HT9

6

C	0.00034300	-1.13919300	0.00000000
H	0.00874600	0.14688000	0.00000000
O	0.00034300	1.35985100	0.00000000
H	-1.05392000	-1.38394200	0.00000000
H	0.52018500	-1.40329300	0.91109300
H	0.52018500	-1.40329300	-0.91109300

TS HT10

5

P	0.00000000	0.24569100	0.00000000
H	0.70361700	-1.04668400	0.00000000
H	1.05247100	-2.29895300	0.00000000
H	-0.87804400	-0.16986600	1.02573000
H	-0.87804400	-0.16986600	-1.02573000

TS HT11

3

Cl	0.00000000	0.00000000	0.00000000
H	0.00000000	0.00000000	1.43913970
O	0.86437469	0.00000000	2.32106356

TS HT12

7

C	0.05344200	-1.19631300	0.00000000
H	0.12640200	0.19798400	0.00000000
N	0.05344200	1.40089400	0.00000000
H	1.10298000	-1.45851400	0.00000000
H	-0.97157100	1.50765300	0.00000000
H	-0.47628100	-1.43775300	0.91173100
H	-0.47628100	-1.43775300	-0.91173100

rev-DSD geometries from Table 5

BHPERI R1

10

H	-0.88933800	1.23994800	-1.14081100
C	0.00000000	0.78409000	-0.69990000
C	0.00000000	-0.78409000	-0.69990000
C	0.00000000	0.67117100	0.81343900
C	0.00000000	-0.67117100	0.81343900
H	0.88933800	-1.23994800	-1.14081100
H	0.00000000	-1.41595200	1.60038800

H	0.88933800	1.23994800	-1.14081100
H	-0.88933800	-1.23994800	-1.14081100
H	0.00000000	1.41595200	1.60038800

TS b1

10

H	1.27301700	-1.44172100	-1.10516200
C	0.40152100	-0.99226500	-0.62626700
C	-0.40152100	0.99226500	-0.62626700
C	0.40152100	-0.55817900	0.73335500
C	-0.40152100	0.55817900	0.73335500
H	-1.27301700	1.44172100	-1.10516200
H	-0.98738100	0.98632400	1.53989800
H	-0.54430000	-1.25769500	-1.07726700
H	0.54430000	1.25769500	-1.07726700
H	0.98738100	-0.98632400	1.53989800

BHPERI P1

10

C	1.84275400	-0.11077100	-0.00000200
C	0.60637600	0.40386500	0.00000200
C	-0.60637600	-0.40386500	0.00000200
H	0.47352100	1.48270500	0.00000300
H	-0.47352100	-1.48270500	0.00000300
C	-1.84275400	0.11077100	-0.00000100

H	-2.00274600	1.18355400	-0.00000500
H	2.71995900	0.52340700	-0.00000200
H	2.00274600	-1.18355400	-0.00000500
H	-2.71995900	-0.52340700	-0.00000300

CRBH R1

8

C	1.06711200	-0.35628800	0.02744700
C	-1.09659400	-0.25354600	0.11379200
O	-0.55726500	1.03239300	-0.12511300
N	0.85640800	0.90121500	0.06249000
H	-1.42013700	-0.35532400	1.15741100
H	-1.90077400	-0.45361100	-0.59085000
H	2.04476800	-0.80751600	0.07410000
O	-0.01046200	-1.16152500	-0.11557800

TS b2

8

C	1.18878600	0.17279700	0.05659200
C	-1.51142200	-0.57461900	0.26464800
O	-1.25146200	0.43616900	-0.37077200
N	0.38323900	1.16845800	0.14877800
H	-0.93220400	-0.86617200	1.14376100
H	-2.35815000	-1.19375600	-0.05146800
H	2.01938400	0.91887600	0.38734900

O 1.31697700 -1.01457100 -0.18529400

CRBH P1

4

O	1.21281500	0.01229700	-0.00000100
H	-1.82074500	0.63530400	-0.00000100
N	-1.16405800	-0.12648800	-0.00000100
C	0.04443900	0.02529000	0.00000200

CRBH R4

8

C	-0.01983800	1.20834800	0.09805900
C	-0.10581400	-1.20379700	0.19680500
O	-1.30456200	-0.56346400	-0.21620800
N	-1.23817300	0.82239400	0.03382200
S	1.23627200	0.00058400	-0.08428700
H	-0.15266800	-1.45043300	1.26166300
H	0.02883100	-2.09373700	-0.41349000
H	0.20111100	2.25847600	0.22415200

TS b3

8

C	0.53610600	0.90733200	0.04937200
C	-1.17508300	-1.06840300	0.22993200

O	-1.71371100	-0.08553300	-0.29173300
N	-0.55374900	1.50338000	0.08660300
S	1.40328000	-0.48420800	-0.06686400
H	-0.93805200	-1.09060800	1.29857800
H	-1.06856700	-2.00061500	-0.33789800
H	0.97392800	1.96558000	0.16097000

CRBH P4

4

H	-2.43180800	0.57139700	0.00000000
N	-1.69356100	-0.11046100	0.00000000
C	-0.49322700	0.01458800	0.00000100
S	1.07788100	0.00714400	0.00000000

CH_2O

4

C	-0.53090900	0.00000000	0.00000000
O	0.67628200	0.00000000	0.00000000
H	-1.11240200	-0.93854600	0.00000100
H	-1.11240000	0.93854700	0.00000100

CADBH R1

3

N	0.00000000	0.00000000	-1.20449800

N	0.00000000	0.00000000	-0.06931700
O	0.00000000	0.00000000	1.11458800

TS b4

9

C	0.77409400	-1.15620000	0.00000000
C	-0.61241800	-1.25132500	0.00000000
O	-1.12824800	0.72531500	0.00000000
N	0.00000000	1.22488000	0.00000000
N	1.10163000	0.80273100	0.00000000
H	1.32112000	-1.33253200	0.91714300
H	1.32112000	-1.33253200	-0.91714300
H	-1.14886100	-1.44279000	0.91946700
H	-1.14886100	-1.44279000	-0.91946700

CADBH P1

9

C	0.43426000	1.10542700	-0.05649000
C	1.15240000	-0.23361800	0.06657300
O	0.08660700	-1.19324400	-0.07101100
N	-1.14111300	-0.48722200	0.02056000
N	-0.99428000	0.72864100	0.04830900
H	0.66903000	1.81700600	0.73261700
H	0.56868900	1.59516200	-1.02221900
H	1.62235200	-0.37242400	1.04180500

H	1.87485500	-0.41457900	-0.72669800

CADBH R4

4

C	0.00000000	0.00000000	-1.18283000
H	0.00000000	0.00000000	-2.24409100
N	0.00000000	0.00000000	-0.01763800
O	0.00000000	0.00000000	1.18306700

TS b5

10

C	0.71356300	-1.31978900	0.00000000
C	-0.65459700	-1.37172200	0.00000000
O	-1.16503100	0.90438300	0.00000000
N	0.00000000	1.25943500	0.00000000
C	1.12682200	0.79168800	0.00000000
H	1.26165900	-1.48377800	0.92045500
H	1.26165900	-1.48377800	-0.92045500
H	-1.21240700	-1.45882600	0.92243900
H	-1.21240700	-1.45882600	-0.92243900
H	2.10701500	1.23303400	0.00000000

CADBH P4

10

C	0.31041400	1.18534400	-0.09470600
C	1.17022200	-0.05889600	0.11507200
O	0.27740200	-1.17556100	-0.11390100
N	-1.04141900	-0.72276400	0.02739700
C	-1.04605600	0.55487300	0.05849900
H	0.50331700	1.96699700	0.63963100
H	0.41560900	1.61933800	-1.09339200
H	1.53745000	-0.12207800	1.14348800
H	2.00055700	-0.15759100	-0.58125100
H	-1.99369400	1.06924500	0.13776400

C_2H_4

6

H	0.00000000	0.92390300	1.23192600
C	0.00000000	0.00000000	0.66586300
C	0.00000000	0.00000000	-0.66586300
H	0.00000000	-0.92390300	-1.23192600
H	0.00000000	-0.92390300	1.23192600
H	0.00000000	0.92390300	-1.23192600

PXBH R1

16

H	-0.56885700	1.70111300	0.00005300
H	1.70111300	0.56885700	-0.00005300
H	1.94101600	-2.07980100	-0.81088800

H	-1.94101600	2.07980100	-0.81088800
H	2.07980100	1.94101600	0.81088800
H	0.56885700	-1.70111300	0.00005300
H	-1.70111300	-0.56885700	-0.00005300
H	-2.07980100	-1.94101600	0.81088800
N	1.58545400	1.58545400	-0.00010100
N	-1.58545400	1.58545400	0.00010100
N	1.58545400	-1.58545400	0.00010100
N	-1.58545400	-1.58545400	-0.00010100
H	1.94092700	-2.07964000	0.81122700
H	2.07964000	1.94092700	-0.81122700
H	-1.94092700	2.07964000	0.81122700
H	-2.07964000	-1.94092700	-0.81122700

TS b6

16

H	1.41516600	0.30742700	0.00000000
H	0.47203800	-1.45181800	0.00000000
H	-1.51896900	0.15313700	0.00000000
H	0.00000000	1.44815700	0.00000000
H	1.49446500	1.85390800	0.82111800
H	1.97444700	-1.68834100	-0.81211100
H	-1.54631000	-1.91818800	0.80075200
H	-2.06887200	1.57118200	-0.81211300
N	1.12021200	1.38964800	0.00000000
N	-1.14883700	-1.42513900	0.00000000

N	1.56138300	-1.24047300	0.00000000
N	-1.54357200	1.26253200	0.00000000
H	-2.06887200	1.57118200	0.81211300
H	-1.54631000	-1.91818800	-0.80075200
H	1.97444700	-1.68834100	0.81211100
H	1.49446500	1.85390800	-0.82111800

BHDIV R2

12

C	-0.35454300	-0.48817800	2.24342900
C	0.35454300	0.48817800	2.24342900
C	1.37619600	2.27050400	-1.30902500
C	0.35454300	1.75015600	-0.93233700
C	-1.37619600	-2.27050400	-1.30902500
C	-0.35454300	-1.75015600	-0.93233700
H	-0.97732600	-1.35114500	2.23026800
H	0.97732600	1.35114500	2.23026800
H	2.27389600	2.73099500	-1.64712300
H	-0.54125600	1.28282900	-0.59555000
H	-2.27389600	-2.73099500	-1.64712300
H	0.54125600	-1.28282900	-0.59555000

TS b7

12

C	0.28006900	1.73863800	0.00110500

C	1.34718200	1.12805100	-0.09165800
C	1.36868900	-1.10193700	0.09280100
C	0.31387200	-1.73300600	-0.00330500
C	-1.64928900	-0.62459300	-0.09502000
C	-1.66085700	0.59283900	0.09607500
H	-0.30584400	2.62717000	0.02683200
H	2.40181000	1.08012700	-0.23080200
H	2.42187700	-1.03358000	0.23427900
H	-0.25469000	-2.63261800	-0.03267500
H	-2.11586300	-1.56605900	-0.26576700
H	-2.14528400	1.52500700	0.26814400

BHDIV P2

12

C	-0.35454300	-0.48817800	2.24342900
C	0.35454300	0.48817800	2.24342900
C	1.37619600	2.27050400	-1.30902500
C	0.35454300	1.75015600	-0.93233700
C	-1.37619600	-2.27050400	-1.30902500
C	-0.35454300	-1.75015600	-0.93233700
H	-0.97732600	-1.35114500	2.23026800
H	0.97732600	1.35114500	2.23026800
H	2.27389600	2.73099500	-1.64712300
H	-0.54125600	1.28282900	-0.59555000
H	-2.27389600	-2.73099500	-1.64712300
H	0.54125600	-1.28282900	-0.59555000

MESS input files

$H^\bullet + CO$

jChS

```
!**************************************************
!              GLOBAL SECTION
!**************************************************
!!!!!!!!!!!!!!!!!!!!!!!!!!!!!!!!!!!!!!!!!!!!!!!!!!!
!
!
TemperatureList[K]                      50 100 200 300 500 700 1000 1500 2000 2500 3000 3500 4000
PressureList[atm]                       0.5 1
!
!
EnergyStepOverTemperature               .2
ExcessEnergyOverTemperature             30
ModelEnergyLimit[kcal/mol]              400
!
CalculationMethod                       direct
!
WellCutoff                              20
ChemicalEigenvalueMax                   0.2
!
ReductionMethod                         diagonalization
!
AtomDistanceMin[bohr]                  1.3
!!
RateOutput                              jChS.out
!
!
!!!!!!!!!!!!!!!!!!!!!!!!!!!!!!!!!!!!!!!!!!!!!!!!!!!
!**************************************************
!               MODEL SECTION
!**************************************************
!!!!!!!!!!!!!!!!!!!!!!!!!!!!!!!!!!!!!!!!!!!!!!!!!!!
!
!
Model
!
  EnergyRelaxation
    Exponential
       Factor[1/cm]                     260
       Power                            0.875
       ExponentCutoff                   10
    End
!
  CollisionFrequency
    LennardJones
       Epsilons[K]                       90.58  617.0
       Sigmas[angstrom]                  3.54    5.62
       Masses[amu]                       39.948 69.0
   End
!
```

```
!**************************************************
!
!****************************************************
!!!!!!!!!!!!!!!!!!!!!!!!!!!!!!!!!!!!!!!!!!!!!!!!!!!!
!****************************************************
!  REACTANTS
!****************************************************


 Bimolecular REACS
 Fragment REACT1
 RRHO
 Geometry[angstrom]               2
 C                 0.00000000    0.00000000   -0.64729900
 O                 0.00000000    0.00000000    0.48547400
  Core  RigidRotor
  SymmetryFactor    1.0000000000000000
  End
     Frequencies[1/cm]            1
2159.29
  ZeroEnergy[kcal/mol]             0.
  ElectronicLevels[1/cm]                      1
   0           1
 !*************************************
 End
!*********
 Fragment REACT2
  Atom
  Name H
  ElectronicLevels[1/cm]                      1
   0       2
 !*************************************
 End
!*********
GroundEnergy[kcal/mol] 0.0
End
 Well HCO
 Species
 RRHO     ! well
 Geometry[angstrom]               3
 H                -0.85891600    1.22453100    0.00000000
 C                 0.06135100    0.58622300    0.00000000
 O                 0.06135100   -0.59273400    0.00000000
  Core  RigidRotor
  SymmetryFactor    1.0000000000000000
  End
     Frequencies[1/cm]            3
1118.88
1898.84
2722.82
  ZeroEnergy[kcal/mol]     -14.72
  ElectronicLevels[1/cm]                      1
   0.0000000000000000        2.0000000000000000
 End
 End
!*********
```

```
 Barrier TS REACS HCO
 RRHO           !
 Geometry[angstrom]            3
 H                 -1.55075700    1.39879200    0.00000000
 C                  0.11076800    0.54977800    0.00000000
 O                  0.11076800   -0.58718200    0.00000000
  Core  RigidRotor
           SymmetryFactor   1
    End
Tunneling    Eckart
    ImaginaryFrequency[1/cm]    786.26
    WellDepth[kcal/mol]   3.67
    WellDepth[kcal/mol]   18.39
    End
     Frequencies[1/cm]            2
     387.45
     2116.64
     ZeroEnergy[kcal/mol]      3.67
     ElectronicLevels[1/cm]                     1
     0.0000000000000000        2.0000000000000000
     End
  End
```

CBS-CVH

```
!****************************************************
!               GLOBAL SECTION
!****************************************************
!!!!!!!!!!!!!!!!!!!!!!!!!!!!!!!!!!!!!!!!!!!!!!!!!!!!
!
!
TemperatureList[K]  50 100 200 300 500 700 1000 1500 2000 2500 3000 3500 4000
PressureList[atm]                       0.5 1
!
!
EnergyStepOverTemperature               .2
ExcessEnergyOverTemperature             30
ModelEnergyLimit[kcal/mol]              400
!
CalculationMethod                       direct
!
WellCutoff                              20
ChemicalEigenvalueMax                   0.2
!
ReductionMethod                         diagonalization
!
AtomDistanceMin[bohr]                  1.3
!!
RateOutput                              HEATlike.out
!
!
!!!!!!!!!!!!!!!!!!!!!!!!!!!!!!!!!!!!!!!!!!!!!!!!!!!!
```

```
!***************************************************
!               MODEL SECTION
!***************************************************
!!!!!!!!!!!!!!!!!!!!!!!!!!!!!!!!!!!!!!!!!!!!!!!!!!!!
!
!
Model
!
  EnergyRelaxation
    Exponential
       Factor[1/cm]                   260
       Power                          0.875
       ExponentCutoff                 10
    End
!
  CollisionFrequency
    LennardJones
       Epsilons[K]                     90.58  617.0
       Sigmas[angstrom]                3.54    5.62
       Masses[amu]                     39.948 69.0
   End
!
!*************************************************
!
!***************************************************
!!!!!!!!!!!!!!!!!!!!!!!!!!!!!!!!!!!!!!!!!!!!!!!!!!!!
!***************************************************
!  REACTANTS
!***************************************************


 Bimolecular REACS
 Fragment REACT1
 RRHO
 Geometry[angstrom]              2
 C                  0.00000000    0.00000000   -0.64729900
 O                  0.00000000    0.00000000    0.48547400
  Core  RigidRotor
  SymmetryFactor    1.0000000000000000
  End
     Frequencies[1/cm]           1
2159.29
  ZeroEnergy[kcal/mol]            0.
  ElectronicLevels[1/cm]                     1
   0            1
 !************************************
 End
!*********
 Fragment REACT2
  Atom
  Name H
  ElectronicLevels[1/cm]                     1
   0       2
 !************************************
 End
!*********
```

```
GroundEnergy[kcal/mol] 0.0
End
 Well HCO
 Species
 RRHO     ! well
 Geometry[angstrom]              3
 H                 -0.85891600    1.22453100    0.00000000
 C                  0.06135100    0.58622300    0.00000000
 O                  0.06135100   -0.59273400    0.00000000
  Core  RigidRotor
  SymmetryFactor    1.0000000000000000
  End
     Frequencies[1/cm]           3
1118.88
1898.84
2722.82
  ZeroEnergy[kcal/mol]     -14.64
  ElectronicLevels[1/cm]                     1
   0.0000000000000000        2.0000000000000000
 End
 End
!*********
 Barrier TS REACS HCO
 RRHO           !
 Geometry[angstrom]           3
 H                 -1.55075700    1.39879200    0.00000000
 C                  0.11076800    0.54977800    0.00000000
 O                  0.11076800   -0.58718200    0.00000000
  Core  RigidRotor
           SymmetryFactor   1
    End
Tunneling    Eckart
    ImaginaryFrequency[1/cm]    786.26
    WellDepth[kcal/mol]   3.70
    WellDepth[kcal/mol]   18.34
    End
     Frequencies[1/cm]           2
     387.45
     2116.64
     ZeroEnergy[kcal/mol]      3.70
     ElectronicLevels[1/cm]                     1
     0.0000000000000000        2.0000000000000000
     End
  End
```

CBS-QB3

```
!****************************************************
!              GLOBAL SECTION
!****************************************************
```

```
!!!!!!!!!!!!!!!!!!!!!!!!!!!!!!!!!!!!!!!!!!!!!!!!!!!!
!
!
TemperatureList[K] 50 100 200 300 500 700 1000 1500 2000 2500 3000 3500 4000
PressureList[atm]                       0.5 1
!
!
EnergyStepOverTemperature               .2
ExcessEnergyOverTemperature             30
ModelEnergyLimit[kcal/mol]              400

CalculationMethod                       direct

WellCutoff                              20
ChemicalEigenvalueMax                   0.2

ReductionMethod                         diagonalization
AtomDistanceMin[bohr]                  1.3
!!
RateOutput                              CBSQB3.out
!
!
!!!!!!!!!!!!!!!!!!!!!!!!!!!!!!!!!!!!!!!!!!!!!!!!!!!!
!***************************************************
!               MODEL SECTION
!***************************************************
!!!!!!!!!!!!!!!!!!!!!!!!!!!!!!!!!!!!!!!!!!!!!!!!!!!!
!
!
Model
!
  EnergyRelaxation
    Exponential
       Factor[1/cm]                     260
       Power                            0.875
       ExponentCutoff                   10
    End
!
  CollisionFrequency
    LennardJones
       Epsilons[K]                       90.58  617.0
       Sigmas[angstrom]                  3.54    5.62
       Masses[amu]                       39.948 69.0
   End
!
!*************************************************
!
!***************************************************
!!!!!!!!!!!!!!!!!!!!!!!!!!!!!!!!!!!!!!!!!!!!!!!!!!!!
!***************************************************
!  REACTANTS
!***************************************************


 Bimolecular REACS
 Fragment REACT1
```

```
 RRHO
 Geometry[angstrom]              2
C        0.000000    0.000000   -0.439519
O        0.000000    0.000000    0.687439
  Core  RigidRotor
  SymmetryFactor    1.0000000000000000
  End
     Frequencies[1/cm]           1
2220.1880
  ZeroEnergy[kcal/mol]            0.
  ElectronicLevels[1/cm]                     1
   0           1
 !************************************
 End
!*********
 Fragment REACT2
  Atom
  Name H
  ElectronicLevels[1/cm]                     1
   0       2
 !************************************
 End
!*********
GroundEnergy[kcal/mol] 0.0
End
 Well HCO
 Species
 RRHO     ! well
 Geometry[angstrom]              3
H   -0.004524    0.000000   -0.010121
C   -0.012232   -0.000000    1.117494
O    0.956706    0.000000    1.780877
  Core  RigidRotor
  SymmetryFactor    1.0000000000000000
  End
     Frequencies[1/cm]           3
1111.0094
1940.9807
2618.5897
  ZeroEnergy[kcal/mol]     -14.42
  ElectronicLevels[1/cm]                     1
   0.0000000000000000        2.0000000000000000
 End
 End
!*********
 Barrier TS REACS HCO
 RRHO           !
 Geometry[angstrom]           3
H      -1.673385    1.508927    0.000000
C       0.201286    0.486214    0.000000
O       0.168499   -0.642581    0.000000

  Core  RigidRotor
          SymmetryFactor   1
    End
Tunneling    Eckart
```

```
    ImaginaryFrequency[1/cm]    401.8416
    WellDepth[kcal/mol]   3.18
    WellDepth[kcal/mol]   17.60
    End
     Frequencies[1/cm]            2
     255.3456
     2190.6226
     ZeroEnergy[kcal/mol]      3.18
     ElectronicLevels[1/cm]                     1
     0.0000000000000000        2.0000000000000000
     End
  End
```

BHPERI1

jChS

```
!***************************************************
!              GLOBAL SECTION
!***************************************************
!!!!!!!!!!!!!!!!!!!!!!!!!!!!!!!!!!!!!!!!!!!!!!!!!!!!
!
!
TemperatureList[K]   200 225 250 275 300 325  350 375 400 425  450 475 500 525 550 575 600 625 650 675 700 750 800 850 900 950 1000 1200 1400 1600 1800 2000
PressureList[atm]                       0.5 1 10 100
!
!
EnergyStepOverTemperature               .2
ExcessEnergyOverTemperature             30
ModelEnergyLimit[kcal/mol]              400
!
CalculationMethod                       direct
!
WellCutoff                              20
ChemicalEigenvalueMax                   0.2
!
ReductionMethod                         diagonalization
!
AtomDistanceMin[bohr]                  1.3
!!
RateOutput                              jChS.out
!
!
!!!!!!!!!!!!!!!!!!!!!!!!!!!!!!!!!!!!!!!!!!!!!!!!!!!!
!***************************************************
!              MODEL SECTION
!***************************************************
!!!!!!!!!!!!!!!!!!!!!!!!!!!!!!!!!!!!!!!!!!!!!!!!!!!!
```

```
!
!
Model
!
  EnergyRelaxation
    Exponential
       Factor[1/cm]                     260
       Power                            0.875
       ExponentCutoff                   10
    End
!
  CollisionFrequency
    LennardJones
       Epsilons[K]                       90.58  617.0
       Sigmas[angstrom]                  3.54    5.62
       Masses[amu]                       39.948 69.0
   End
!
!**************************************************
!
!****************************************************
!!!!!!!!!!!!!!!!!!!!!!!!!!!!!!!!!!!!!!!!!!!!!!!!!!!!
!****************************************************
!  REACTANTS
!****************************************************
 Well PROD_OPEN
 Species
 RRHO     ! well
 Geometry[angstrom]               10
 H                  0.09014100   -2.59586800   -0.43089500
 C                 -0.11062500   -1.53355200   -0.49117800
 C                  0.11062500    1.53355200   -0.49117800
 C                  0.11062500   -0.72689500    0.55277800
 C                 -0.11062500    0.72689500    0.55277800
 H                 -0.09014100    2.59586800   -0.43089500
 H                 -0.46064100    1.16147500    1.48541400
 H                 -0.51010600   -1.15189600   -1.42411600
 H                  0.51010600    1.15189600   -1.42411600
 H                  0.46064100   -1.16147500    1.48541400
  Core  RigidRotor
  SymmetryFactor    2.0000000000000000
  End
     Frequencies[1/cm]            24
170.10
272.90
467.36
620.96
756.62
891.37
947.55
948.99
1015.80
1035.56
1068.60
1106.93
1311.72
```

```
1342.25
1443.83
1474.01
1680.75
1687.83
3148.39
3152.66
3156.81
3167.66
3245.09
3246.62
  ZeroEnergy[kcal/mol]     -9.41
  ElectronicLevels[1/cm]                     1
   0.0000000000000000        1.0000000000000000
 End
 End

 Well REAC_CLOSE
 Species
 RRHO     ! well
 Geometry[angstrom]              10
 H                -0.88924800    1.24031000   -1.14101400
 C                 0.00000000    0.78411900   -0.69986400
 C                 0.00000000   -0.78411900   -0.69986400
 C                 0.00000000    0.67116200    0.81344400
 C                 0.00000000   -0.67116200    0.81344400
 H                 0.88924800   -1.24031000   -1.14101400
 H                 0.00000000   -1.41596600    1.60054700
 H                 0.88924800    1.24031000   -1.14101400
 H                -0.88924800   -1.24031000   -1.14101400
 H                 0.00000000    1.41596600    1.60054700
  Core  RigidRotor
  SymmetryFactor    2.0000000000000000
  End
     Frequencies[1/cm]           24
314.78
649.95
862.03
872.09
902.35
908.73
938.80
1007.03
1041.49
1107.07
1140.87
1182.60
1224.13
1245.15
1330.27
1476.24
1497.72
1618.65
3056.87
3062.55
3101.07
```

```
3115.22
3183.69
3215.37
  ZeroEnergy[kcal/mol]     0.
  ElectronicLevels[1/cm]                     1
   0.0000000000000000        1.0000000000000000
 End
 End
!*********
 Barrier TS REAC_CLOSE PROD_OPEN
 RRHO           !
 Geometry[angstrom]           10
 H                  1.27409200   -1.44251600   -1.10412100
 C                  0.40274700   -0.99171600   -0.62582600
 C                 -0.40274700    0.99171600   -0.62582600
 C                  0.40274700   -0.55749800    0.73298600
 C                 -0.40274700    0.55749800    0.73298600
 H                 -1.27409200    1.44251600   -1.10412100
 H                 -0.99058900    0.98389500    1.53917500
 H                 -0.54267000   -1.25670400   -1.07801000
 H                  0.54267000    1.25670400   -1.07801000
 H                  0.99058900   -0.98389500    1.53917500
  Core  RigidRotor
          SymmetryFactor   2
    End
Tunneling    Eckart
    ImaginaryFrequency[1/cm]    772.41
    WellDepth[kcal/mol]   33.16
    WellDepth[kcal/mol]   42.56
    End
     Frequencies[1/cm]           23
     468.21
651.10
686.76
736.38
889.19
896.86
925.61
954.15
1013.63
1035.60
1126.18
1171.08
1248.29
1390.19
1520.16
1527.47
1539.91
3096.35
3096.48
3169.26
3189.54
3226.30
3226.71
     ZeroEnergy[kcal/mol]      33.16
     ElectronicLevels[1/cm]                     1
```

```
    0.0000000000000000        1.0000000000000000
    End
  End
```

W3lite-F12

```
!***************************************************
!              GLOBAL SECTION
!***************************************************
!!!!!!!!!!!!!!!!!!!!!!!!!!!!!!!!!!!!!!!!!!!!!!!!!!!!
!
!
TemperatureList[K]    200 225 250 275 300 325  350 375 400 425  450 475 500 525 550 575 600 625 650 675 700 750 800 850 900 950 1000 1200 1400 1600 1800 2000
PressureList[atm]                      0.5 1 10 100
!
!
EnergyStepOverTemperature              .2
ExcessEnergyOverTemperature            30
ModelEnergyLimit[kcal/mol]             400
!
CalculationMethod                      direct
!
WellCutoff                             20
ChemicalEigenvalueMax                  0.2
!
ReductionMethod                        diagonalization
!
AtomDistanceMin[bohr]                 1.3
!!
RateOutput                             Karton.out
!
!
!!!!!!!!!!!!!!!!!!!!!!!!!!!!!!!!!!!!!!!!!!!!!!!!!!!!
!***************************************************
!              MODEL SECTION
!***************************************************
!!!!!!!!!!!!!!!!!!!!!!!!!!!!!!!!!!!!!!!!!!!!!!!!!!!!
!
!
Model
!
  EnergyRelaxation
    Exponential
       Factor[1/cm]                    260
       Power                           0.875
       ExponentCutoff                  10
    End
!
  CollisionFrequency
    LennardJones
```

```
      Epsilons[K]                      90.58  617.0
      Sigmas[angstrom]                 3.54    5.62
      Masses[amu]                      39.948 69.0
  End
!
!**************************************************
!
!****************************************************
!!!!!!!!!!!!!!!!!!!!!!!!!!!!!!!!!!!!!!!!!!!!!!!!!!!!
!****************************************************
!  REACTANTS
!****************************************************
 Well PROD_OPEN
 Species
 RRHO     ! well
 Geometry[angstrom]               10
 H                  0.09014100   -2.59586800   -0.43089500
 C                 -0.11062500   -1.53355200   -0.49117800
 C                  0.11062500    1.53355200   -0.49117800
 C                  0.11062500   -0.72689500    0.55277800
 C                 -0.11062500    0.72689500    0.55277800
 H                 -0.09014100    2.59586800   -0.43089500
 H                 -0.46064100    1.16147500    1.48541400
 H                 -0.51010600   -1.15189600   -1.42411600
 H                  0.51010600    1.15189600   -1.42411600
 H                  0.46064100   -1.16147500    1.48541400
  Core  RigidRotor
  SymmetryFactor    2.0000000000000000
  End
     Frequencies[1/cm]            24
170.10
272.90
467.36
620.96
756.62
891.37
947.55
948.99
1015.80
1035.56
1068.60
1106.93
1311.72
1342.25
1443.83
1474.01
1680.75
1687.83
3148.39
3152.66
3156.81
3167.66
3245.09
3246.62
  ZeroEnergy[kcal/mol]     0.
  ElectronicLevels[1/cm]                     1
```

```
  0.0000000000000000        1.0000000000000000
 End
 End

 Well REAC_CLOSE
 Species
 RRHO     ! well
 Geometry[angstrom]              10
 H                -0.88681500    1.24201700   -1.14058200
 C                 0.00000000    0.78467600   -0.69862500
 C                 0.00000000   -0.78467600   -0.69862500
 C                 0.00000000    0.66758300    0.81245700
 C                 0.00000000   -0.66758300    0.81245700
 H                 0.88681500   -1.24201700   -1.14058200
 H                 0.00000000   -1.41114900    1.59816800
 H                 0.88681500    1.24201700   -1.14058200
 H                -0.88681500   -1.24201700   -1.14058200
 H                 0.00000000    1.41114900    1.59816800
  Core  RigidRotor
  SymmetryFactor    2.0000000000000000
  End
     Frequencies[1/cm]            24
314.78
649.95
862.03
872.09
902.35
908.73
938.80
1007.03
1041.49
1107.07
1140.87
1182.60
1224.13
1245.15
1330.27
1476.24
1497.72
1618.65
3056.87
3062.55
3101.07
3115.22
3183.69
3215.37
  ZeroEnergy[kcal/mol]     0.
  ElectronicLevels[1/cm]                     1
   0.0000000000000000        1.0000000000000000
 End
 End
!*********
 Barrier TS REAC_CLOSE PROD_OPEN
 RRHO           !
 Geometry[angstrom]           10
 H                 1.25355500   -1.44515000   -1.10420900
```

```
 C                 0.38407400   -0.99466100   -0.62685100
 C                -0.38407400    0.99466100   -0.62685100
 C                 0.38407400   -0.56603600    0.73361200
 C                -0.38407400    0.56603600    0.73361200
 H                -1.25355500    1.44515000   -1.10420900
 H                -0.95204700    1.01049400    1.54226800
 H                -0.55574500   -1.27074700   -1.07862800
 H                 0.55574500    1.27074700   -1.07862800
 H                 0.95204700   -1.01049400    1.54226800
  Core  RigidRotor
           SymmetryFactor   2
    End
Tunneling    Eckart
    ImaginaryFrequency[1/cm]    772.41
    WellDepth[kcal/mol]   33.20
    WellDepth[kcal/mol]   33.20
    End
     Frequencies[1/cm]            23
     468.21
651.10
686.76
736.38
889.19
896.86
925.61
954.15
1013.63
1035.60
1126.18
1171.08
1248.29
1390.19
1520.16
1527.47
1539.91
3096.35
3096.48
3169.26
3189.54
3226.30
3226.71
     ZeroEnergy[kcal/mol]      33.20
     ElectronicLevels[1/cm]                      1
     0.0000000000000000        1.0000000000000000
     End
  End
```

CBS-QB3

```
!****************************************************
```

```
!              GLOBAL SECTION
!**************************************************
!!!!!!!!!!!!!!!!!!!!!!!!!!!!!!!!!!!!!!!!!!!!!!!!!!!
!
!
TemperatureList[K]     200 225 250 275 300 325  350 375 400 425  450 475 500 525 550 575 600 625 650 675 700 750 800 850 900 950 1000 1200 1400 1600 1800 2000
PressureList[atm]                       0.5 1 10 100
!
!
EnergyStepOverTemperature               .2
ExcessEnergyOverTemperature             30
ModelEnergyLimit[kcal/mol]              400
!
CalculationMethod                       direct
!
WellCutoff                              20
ChemicalEigenvalueMax                   0.2
!
ReductionMethod                         diagonalization
!
AtomDistanceMin[bohr]                  1.3
!!
RateOutput                              CBSQB3.out
!
!
!!!!!!!!!!!!!!!!!!!!!!!!!!!!!!!!!!!!!!!!!!!!!!!!!!!
!**************************************************
!             MODEL SECTION
!**************************************************
!!!!!!!!!!!!!!!!!!!!!!!!!!!!!!!!!!!!!!!!!!!!!!!!!!!
!
!
Model
!
  EnergyRelaxation
    Exponential
       Factor[1/cm]                     260
       Power                            0.875
       ExponentCutoff                   10
    End
!
  CollisionFrequency
    LennardJones
       Epsilons[K]                       90.58  617.0
       Sigmas[angstrom]                  3.54    5.62
       Masses[amu]                       39.948 69.0
   End
!
!************************************************
!
!**************************************************
!!!!!!!!!!!!!!!!!!!!!!!!!!!!!!!!!!!!!!!!!!!!!!!!!!!
!**************************************************
!  REACTANTS
!**************************************************
 Well REAC_OPEN
```

```
 Species
 RRHO     ! well
 Geometry[angstrom]               10
 H                  0.08687000   -2.60781500   -0.41351600
 C                 -0.09972500   -1.54300300   -0.48926600
 C                  0.09972500    1.54300300   -0.48926600
 C                  0.09972500   -0.72766100    0.54889400
 C                 -0.09972500    0.72766100    0.54889400
 H                 -0.08687000    2.60781500   -0.41351600
 H                 -0.41569100    1.15953600    1.49655400
 H                 -0.46881200   -1.17501500   -1.44080700
 H                  0.46881200    1.17501500   -1.44080700
 H                  0.41569100   -1.15953600    1.49655400
  Core  RigidRotor
  SymmetryFactor    2.0000000000000000
  End
     Frequencies[1/cm]            24
162.53 274.81 473.28 619.75 757.49 883.62
943.63 945.02 1018.93 1033.03 1068.72 1105.98
1311.61 1343.79 1437.80 1463.52 1673.74 1695.09
3118.16 3128.83 3133.92 3139.64 3217.68 3219.63
  ZeroEnergy[kcal/mol]     0.
  ElectronicLevels[1/cm]                      1
   0.0000000000000000        1.0000000000000000
 End
 End

 Well PROD_CLOSE
 Species
 RRHO     ! well
 Geometry[angstrom]               10
 H                 -0.88863500    1.24565400   -1.14339500
 C                  0.00000000    0.78638500   -0.70000000
 C                  0.00000000   -0.78638500   -0.70000000
 C                  0.00000000    0.66864600    0.81426400
 C                  0.00000000   -0.66864600    0.81426400
 H                  0.88863500   -1.24565400   -1.14339500
 H                  0.00000000   -1.41442700    1.60121000
 H                  0.88863500    1.24565400   -1.14339500
 H                 -0.88863500   -1.24565400   -1.14339500
 H                  0.00000000    1.41442700    1.60121000
  Core  RigidRotor
  SymmetryFactor    2.0000000000000000
  End
     Frequencies[1/cm]            24
323.85 650.74 865.67 868.08 886.30 897.37
940.68 997.79 1039.73 1099.04 1129.89 1169.32
1214.61 1235.80 1317.40 1469.09 1488.90 1635.56
3027.58 3033.12 3063.30 3077.99 3162.14 3193.97
  ZeroEnergy[kcal/mol]     9.76
  ElectronicLevels[1/cm]                      1
   0.0000000000000000        1.0000000000000000
 End
 End
!*********
 Barrier TS REAC_OPEN PROD_CLOSE
```

```
 RRHO            !
 Geometry[angstrom]            10
 H                  1.26136400   -1.45280700   -1.10161500
 C                  0.39035400   -0.99502000   -0.62651600
 C                 -0.39035400    0.99502000   -0.62651600
 C                  0.39035400   -0.56452900    0.73348100
 C                 -0.39035400    0.56452900    0.73348100
 H                 -1.26136400    1.45280700   -1.10161500
 H                 -0.96838000    1.00190500    1.54234100
 H                 -0.55034900   -1.27028000   -1.08251700
 H                  0.55034900    1.27028000   -1.08251700
 H                  0.96838000   -1.00190500    1.54234100
  Core  RigidRotor
          SymmetryFactor   2
    End
Tunneling    Eckart
    ImaginaryFrequency[1/cm]    736.27
    WellDepth[kcal/mol]   41.79
    WellDepth[kcal/mol]   32.03
    End
     Frequencies[1/cm]            23
     471.50 650.21 699.95 742.79 892.64 898.73
     930.18 957.37 1013.46 1030.91 1122.30 1164.77
     1244.74 1384.76 1512.78 1519.63 1543.75 3069.37
     3069.63 3139.20 3159.86 3205.60 3206.58
     ZeroEnergy[kcal/mol]      41.79
     ElectronicLevels[1/cm]                      1
     0.0000000000000000        1.0000000000000000
     End
  End
```

CRBH4

jChS

```
!***************************************************
!               GLOBAL SECTION
!***************************************************
!!!!!!!!!!!!!!!!!!!!!!!!!!!!!!!!!!!!!!!!!!!!!!!!!!!!
!
!
TemperatureList[K]    200 225 250 275 300 325  350 375 400 425  450 475 500 525 550 575 600 625 650 675 700 750 800 850 900 950 1000 1200 1400 1600 1800 2000
PressureList[atm]                       0.5 1 10 100
!
!
EnergyStepOverTemperature               .2
ExcessEnergyOverTemperature             30
ModelEnergyLimit[kcal/mol]              400
```

```
!
CalculationMethod                       direct
!
WellCutoff                              20
ChemicalEigenvalueMax                   0.2
!
ReductionMethod                         diagonalization
!
AtomDistanceMin[bohr]                  1.3
!!
RateOutput                              jChS.out
!
!
!!!!!!!!!!!!!!!!!!!!!!!!!!!!!!!!!!!!!!!!!!!!!!!!!!!!
!***************************************************
!              MODEL SECTION
!***************************************************
!!!!!!!!!!!!!!!!!!!!!!!!!!!!!!!!!!!!!!!!!!!!!!!!!!!!
!
!
Model
!
  EnergyRelaxation
    Exponential
       Factor[1/cm]                     260
       Power                            0.875
       ExponentCutoff                   10
    End
!
  CollisionFrequency
    LennardJones
       Epsilons[K]                       90.58  617.0
       Sigmas[angstrom]                  3.54    5.62
       Masses[amu]                       39.948 69.0
   End
!
!*************************************************
!
!***************************************************
!!!!!!!!!!!!!!!!!!!!!!!!!!!!!!!!!!!!!!!!!!!!!!!!!!!!
!***************************************************
!  REACTANTS
!***************************************************
 Well REAC_CLOSE
 Species
 RRHO     ! well
 Geometry[angstrom]              8
 C                -0.02003100    1.20888100    0.09746300
 C                -0.10577400   -1.20488100    0.19540400
 O                -1.30542900   -0.56334400   -0.21480400
 N                -1.23844900    0.82283300    0.03373200
 S                 1.23690200    0.00076400   -0.08372100
 H                -0.15182500   -1.45533700    1.25953400
 H                 0.02811200   -2.09328300   -0.41762100
 H                 0.20068500    2.25932500    0.22272800
  Core  RigidRotor
```

```
  SymmetryFactor    1.0000000000000000
  End
     Frequencies[1/cm]            18
200.42
482.41
524.75
678.99
784.64
837.75
842.42
891.51
981.53
996.21
1214.28
1233.98
1338.13
1500.31
1584.01
3042.71
3158.81
3246.18
  ZeroEnergy[kcal/mol]     0.
  ElectronicLevels[1/cm]                       1
   0.0000000000000000        1.0000000000000000
 End
 End

 Well PROD_OPEN
 Species
 RRHO     ! well
 Geometry[angstrom]               8
  C                 -0.02003100    1.20888100    0.09746300
 C                 -0.10577400   -1.20488100    0.19540400
 O                 -1.30542900   -0.56334400   -0.21480400
 N                 -1.23844900    0.82283300    0.03373200
 S                  1.23690200    0.00076400   -0.08372100
 H                 -0.15182500   -1.45533700    1.25953400
 H                  0.02811200   -2.09328300   -0.41762100
 H                  0.20068500    2.25932500    0.22272800
  Core  RigidRotor
  SymmetryFactor    1.0000000000000000
  End
     Frequencies[1/cm]            18
200.42
482.41
524.75
678.99
784.64
837.75
842.42
891.51
981.53
996.21
1214.28
1233.98
1338.13
```

```
1500.31
1584.01
3042.71
3158.81
3246.18
  ZeroEnergy[kcal/mol]     0.
  ElectronicLevels[1/cm]                     1
   0.0000000000000000        1.0000000000000000
 End
 End
!*********
 Barrier TS REAC_CLOSE PROD_OPEN
 RRHO           !
 Geometry[angstrom]            8
 C                 0.53937900    0.90635200    0.04997800
 C                -1.18561800   -1.06690900    0.22990500
 O                -1.71476700   -0.07944500   -0.29278100
 N                -0.55193700    1.50114300    0.08705600
 S                 1.40708400   -0.48628900   -0.06684700
 H                -0.94611600   -1.08967800    1.29798200
 H                -1.08921000   -2.00070800   -0.33725500
 H                 0.98111600    1.96190800    0.16239100
  Core  RigidRotor
           SymmetryFactor   1
    End
Tunneling    Eckart
    ImaginaryFrequency[1/cm]    713.47
    WellDepth[kcal/mol]   42.06
    WellDepth[kcal/mol]   42.06
    End
     Frequencies[1/cm]            17
149.17
210.79
349.28
468.32
499.70
581.09
615.17
648.09
816.47
1110.21
1238.50
1481.70
1635.27
1763.29
2705.60
3016.87
3109.01
     ZeroEnergy[kcal/mol]      42.06
     ElectronicLevels[1/cm]                     1
     0.0000000000000000        1.0000000000000000
     End
  End
```

W3lite-F12

```
!****************************************************
!              GLOBAL SECTION
!****************************************************
!!!!!!!!!!!!!!!!!!!!!!!!!!!!!!!!!!!!!!!!!!!!!!!!!!!!!!
!
!
TemperatureList[K]   200 225 250 275 300 325  350 375 400 425  450 475 500 525 550 575 600 625 650 675 700 750 800 850 900 950 1000 1200 1400 1600 1800 2000
PressureList[atm]                       0.5 1 10 100
!
!
EnergyStepOverTemperature               .2
ExcessEnergyOverTemperature             30
ModelEnergyLimit[kcal/mol]              400
!
CalculationMethod                       direct
!
WellCutoff                              20
ChemicalEigenvalueMax                   0.2
!
ReductionMethod                         diagonalization
AtomDistanceMin[bohr]                  1.3
!!
RateOutput                              Karton.out
!
!
!!!!!!!!!!!!!!!!!!!!!!!!!!!!!!!!!!!!!!!!!!!!!!!!!!!!!!
!****************************************************
!              MODEL SECTION
!****************************************************
!!!!!!!!!!!!!!!!!!!!!!!!!!!!!!!!!!!!!!!!!!!!!!!!!!!!!!
!
!
Model
!
  EnergyRelaxation
    Exponential
       Factor[1/cm]                     260
       Power                            0.875
       ExponentCutoff                   10
    End
!
  CollisionFrequency
    LennardJones
       Epsilons[K]                       90.58  617.0
       Sigmas[angstrom]                  3.54    5.62
       Masses[amu]                       39.948 69.0
   End
!
!**************************************************
!
```

```
!***************************************************
!!!!!!!!!!!!!!!!!!!!!!!!!!!!!!!!!!!!!!!!!!!!!!!!!!!!
!***************************************************
!  REACTANTS
!***************************************************
 Well REAC_CLOSE
 Species
 RRHO     ! well
 Geometry[angstrom]               8
C -0.0211260 1.2143720 0.0861400
C -0.1086300 -1.2218440 0.1704460
O -1.3154940 -0.5575770 -0.1896810
N -1.2315760 0.8329400 0.0324420
S 1.2396090 0.0010990 -0.0736110
H -0.1370880 -1.5274350 1.2190290
H 0.0170930 -2.0785540 -0.4853320
H 0.2097690 2.2632700 0.1949200
  Core  RigidRotor
  SymmetryFactor    1.0000000000000000
  End
     Frequencies[1/cm]            18
200.42
482.41
524.75
678.99
784.64
837.75
842.42
891.51
981.53
996.21
1214.28
1233.98
1338.13
1500.31
1584.01
3042.71
3158.81
3246.18
  ZeroEnergy[kcal/mol]     0.
  ElectronicLevels[1/cm]                      1
   0.0000000000000000        1.0000000000000000
 End
 End

 Well PROD_OPEN
 Species
 RRHO     ! well
 Geometry[angstrom]               8
C -0.0211260 1.2143720 0.0861400
C -0.1086300 -1.2218440 0.1704460
O -1.3154940 -0.5575770 -0.1896810
N -1.2315760 0.8329400 0.0324420
S 1.2396090 0.0010990 -0.0736110
H -0.1370880 -1.5274350 1.2190290
H 0.0170930 -2.0785540 -0.4853320
```

```
H 0.2097690 2.2632700 0.1949200
  Core  RigidRotor
  SymmetryFactor    1.0000000000000000
  End
     Frequencies[1/cm]            18
200.42
482.41
524.75
678.99
784.64
837.75
842.42
891.51
981.53
996.21
1214.28
1233.98
1338.13
1500.31
1584.01
3042.71
3158.81
3246.18
  ZeroEnergy[kcal/mol]     0.
  ElectronicLevels[1/cm]                       1
   0.0000000000000000        1.0000000000000000
 End
 End
!*********
 Barrier TS REAC_CLOSE PROD_OPEN
 RRHO            !
 Geometry[angstrom]            8
C 0.6112370 0.8781110 0.0482300
C -1.3356260 -1.0194430 0.2278640
O -1.7615960 0.0024910 -0.2939760
N -0.4252580 1.5553560 0.0890100
S 1.4144820 -0.5569100 -0.0651010
H -1.0756240 -1.0632920 1.2908480
H -1.3035850 -1.9597990 -0.3387530
H 1.1634070 1.8742220 0.1616990
  Core  RigidRotor
           SymmetryFactor   1
    End
Tunneling    Eckart
    ImaginaryFrequency[1/cm]    713.47
    WellDepth[kcal/mol]   40.40
    WellDepth[kcal/mol]   40.40
    End
     Frequencies[1/cm]            17
149.17
210.79
349.28
468.32
499.70
581.09
615.17
```

```
648.09
816.47
1110.21
1238.50
1481.70
1635.27
1763.29
2705.60
3016.87
3109.01
     ZeroEnergy[kcal/mol]      40.40
     ElectronicLevels[1/cm]                      1
     0.0000000000000000        1.0000000000000000
     End
  End
```

CBS-QB3

```
!***************************************************
!              GLOBAL SECTION
!***************************************************
!!!!!!!!!!!!!!!!!!!!!!!!!!!!!!!!!!!!!!!!!!!!!!!!!!!!
!
!
TemperatureList[K]   200 225 250 275 300 325  350 375 400 425  450 475 500 525 550 575 600 625 650 675 700 750 800 850 900 950 1000 1200 1400 1600 1800 2000
PressureList[atm]                       0.5 1 10 100
!
!
EnergyStepOverTemperature              .2
ExcessEnergyOverTemperature            30
ModelEnergyLimit[kcal/mol]             400
!
CalculationMethod                       direct
!
WellCutoff                             20
ChemicalEigenvalueMax                  0.2
!
ReductionMethod                         diagonalization
!
AtomDistanceMin[bohr]                 1.3
!!
RateOutput                              CBSQB3.out
!
!
!!!!!!!!!!!!!!!!!!!!!!!!!!!!!!!!!!!!!!!!!!!!!!!!!!!!
!***************************************************
!              MODEL SECTION
!***************************************************
!!!!!!!!!!!!!!!!!!!!!!!!!!!!!!!!!!!!!!!!!!!!!!!!!!!!
!
```

```
!
Model
!
  EnergyRelaxation
    Exponential
       Factor[1/cm]                    260
       Power                           0.875
       ExponentCutoff                  10
    End
!
  CollisionFrequency
    LennardJones
       Epsilons[K]                      90.58  617.0
       Sigmas[angstrom]                 3.54    5.62
       Masses[amu]                      39.948 69.0
   End
!
!**************************************************
!
!****************************************************
!!!!!!!!!!!!!!!!!!!!!!!!!!!!!!!!!!!!!!!!!!!!!!!!!!!!
!****************************************************
!  REACTANTS
!****************************************************
 Well REAC_CLOSE
 Species
 RRHO     ! well
 Geometry[angstrom]              8
 C                -0.02928800    1.21849000    0.08546900
 C                -0.10614600   -1.22508200    0.16950700
 O                -1.31591700   -0.56038300   -0.18814800
 N                -1.23878600    0.82698400    0.03157200
 S                 1.24518900    0.00477200   -0.07300900
 H                -0.13019700   -1.53357800    1.21944500
 H                 0.01958900   -2.07884900   -0.49316700
 H                 0.19901900    2.26980600    0.19619700
  Core  RigidRotor
  SymmetryFactor    1.0000000000000000
  End
     Frequencies[1/cm]           18
168.97
479.75
521.55
645.50
763.59
825.51
830.77
890.45
960.90
971.36
1199.34
1228.19
1333.46
1495.04
1615.51
3022.61
```

```
3137.17
3224.14
  ZeroEnergy[kcal/mol]     0.
  ElectronicLevels[1/cm]                     1
   0.0000000000000000        1.0000000000000000
 End
 End

 Well PROD_OPEN
 Species
 RRHO     ! well
 Geometry[angstrom]               8
 C                 -0.02928800    1.21849000    0.08546900
 C                 -0.10614600   -1.22508200    0.16950700
 O                 -1.31591700   -0.56038300   -0.18814800
 N                 -1.23878600    0.82698400    0.03157200
 S                  1.24518900    0.00477200   -0.07300900
 H                 -0.13019700   -1.53357800    1.21944500
 H                  0.01958900   -2.07884900   -0.49316700
 H                  0.19901900    2.26980600    0.19619700
  Core  RigidRotor
  SymmetryFactor    1.0000000000000000
  End
     Frequencies[1/cm]            18
168.97
479.75
521.55
645.50
763.59
825.51
830.77
890.45
960.90
971.36
1199.34
1228.19
1333.46
1495.04
1615.51
3022.61
3137.17
3224.14
  ZeroEnergy[kcal/mol]     0.
  ElectronicLevels[1/cm]                     1
   0.0000000000000000        1.0000000000000000
 End
 End
!*********
 Barrier TS REAC_CLOSE PROD_OPEN
 RRHO            !
 Geometry[angstrom]            8
 C                  0.62540400    0.86937200    0.05343600
 C                 -1.40090400   -1.00407800    0.23980900
 O                 -1.77069800    0.02065800   -0.31383200
 N                 -0.41894900    1.54383800    0.09299400
 S                  1.44466900   -0.56454400   -0.06828200
```

```
 H                 -1.09770700   -1.02370500    1.29457800
 H                 -1.44615500   -1.96860400   -0.29007000
 H                  1.18038200    1.86112200    0.18824100
  Core  RigidRotor
           SymmetryFactor   1
    End
Tunneling    Eckart
    ImaginaryFrequency[1/cm]    477.57
    WellDepth[kcal/mol]   40.96
    WellDepth[kcal/mol]   40.96
    End
     Frequencies[1/cm]            17
     132.98
183.66
289.22
409.57
456.92
495.68
602.17
629.43
795.76
1133.67
1239.93
1498.06
1690.59
1724.18
2719.43
2965.64
3055.29
     ZeroEnergy[kcal/mol]      40.96
     ElectronicLevels[1/cm]                       1
     0.0000000000000000        1.0000000000000000
     End
  End
```

$H_2S + Cl^{\bullet}$

jChS

```
!***************************************************
!               GLOBAL SECTION
!***************************************************
!!!!!!!!!!!!!!!!!!!!!!!!!!!!!!!!!!!!!!!!!!!!!!!!!!!!
!
!
TemperatureList[K]                      200 225 250 275 300 325 350 375 400 425 430 450 475 500 600 700 800 900 1000
PressureList[atm]                       0.5 1
!
```

```
!
EnergyStepOverTemperature              .2
ExcessEnergyOverTemperature            30
ModelEnergyLimit[kcal/mol]             400
!
CalculationMethod                      direct
!
WellCutoff                             20
ChemicalEigenvalueMax                  0.2
!
ReductionMethod                        diagonalization
!
AtomDistanceMin[bohr]                 1.3
!!
RateOutput                             junchs.out
!
!
!!!!!!!!!!!!!!!!!!!!!!!!!!!!!!!!!!!!!!!!!!!!!!!!!!!!
!***************************************************
!               MODEL SECTION
!***************************************************
!!!!!!!!!!!!!!!!!!!!!!!!!!!!!!!!!!!!!!!!!!!!!!!!!!!!
!
!
Model
!
  EnergyRelaxation
    Exponential
       Factor[1/cm]                    260
       Power                           0.875
       ExponentCutoff                  10
    End
!
  CollisionFrequency
    LennardJones
       Epsilons[K]                      90.58  617.0
       Sigmas[angstrom]                 3.54    5.62
       Masses[amu]                      39.948 69.0
   End
!
!*************************************************
!
!***************************************************
!!!!!!!!!!!!!!!!!!!!!!!!!!!!!!!!!!!!!!!!!!!!!!!!!!!!
!***************************************************
!  REACTANTS
!***************************************************


 Bimolecular REACS
 Fragment REACT1
 RRHO
 Geometry[angstrom]              3
 S                  0.00000000    0.00000000    0.10283933
 H                  0.00000000    0.96667700   -0.82271467
 H                  0.00000000   -0.96667700   -0.82271467
```

```
  Core  RigidRotor
  SymmetryFactor    2.0000000000000000
  End
     Frequencies[1/cm]           3
   1217.90 2736.90 2751.70
  ZeroEnergy[kJ/mol]            0.
  ElectronicLevels[1/cm]                     1
   0           1
 !************************************
 End
!*********
 Fragment REACT2
  Atom
  Name Cl
  ElectronicLevels[1/cm]                     2
   0       4
   881.00  2

 !************************************
 End
!*********
GroundEnergy[kJ/mol] 0.0
End
 Bimolecular PRODS
 Fragment PROD1
 RRHO
 Geometry[angstrom]              2
   H                  0.00000000    0.00000000   -1.26323400
   S                  0.00000000    0.00000000    0.07895200
  Core  RigidRotor
  SymmetryFactor    1.0000000000000000
  End
     Frequencies[1/cm]           1
   2719.97
  ZeroEnergy[kJ/mol]            0.
  ElectronicLevels[1/cm]                     2
   0.0000000000000000        2.0000000000000000
   376.95999999999998        2.0000000000000000
 !************************************
 End
!*********
 Fragment PROD2
 RRHO
 Geometry[angstrom]              2
   H                  0.00000000    0.00000000   -1.20561900
   Cl                 0.00000000    0.00000000    0.07091900
  Core  RigidRotor
  SymmetryFactor    1.0000000000000000
  End
     Frequencies[1/cm]           1
   3006.52
  ZeroEnergy[kJ/mol]            0.
  ElectronicLevels[1/cm]                     1
   0.0000000000000000        1.0000000000000000

 !************************************
```

```
 End
GroundEnergy[kJ/mol]   -53.18
End
!*********
 Well WR
 Species
 RRHO     ! well
 Geometry[angstrom]              4
   H                  0.86853200   -1.21794200    0.97069800
   S                 -0.05263800   -1.26295000    0.00000000
   H                  0.86853200   -1.21794200   -0.97069800
   Cl                -0.05263800    1.33194700    0.00000000
  Core  RigidRotor
  SymmetryFactor    1.0000000000000000
  End
     Frequencies[1/cm]           6
   235.51 361.29 394.61 1211.09 2735.17 2749.91
  ZeroEnergy[kJ/mol]     -38.35
  ElectronicLevels[1/cm]                     1
   0.0000000000000000        2.0000000000000000
 End
 End
 !************************************
 Well WP
 Species
 RRHO     ! well
 Geometry[angstrom]              4
    S                 -1.89837200   -0.07864900   -0.00000300
    H                 -1.96120000    1.26293700   -0.00000900
    H                  0.58255000   -0.07505800    0.00001500
    Cl                 1.86780000    0.00414800    0.00000200
   Core  RigidRotor
   SymmetryFactor    1.0000000000000000
  End
    Frequencies[1/cm]           6
 92.55 144.41 254.63 397.12 2715.09 2844.57
  ZeroEnergy[kJ/mol]             -61.64
  ElectronicLevels[1/cm]                     1
   0.0000000000000000        2.0000000000000000
 End
 End
 !************************************
 Barrier B1  REACS WR
   RRHO
     Stoichiometry  H2S1Cl1
     Core PhaseSpaceTheory
       FragmentGeometry[angstrom]              3
 S                  0.00000000    0.00000000    0.10283933
 H                  0.00000000    0.96667700   -0.82271467
 H                  0.00000000   -0.96667700   -0.82271467
       FragmentGeometry[angstrom]              1
 Cl                   0. 0. 0.
  SymmetryFactor     2.000000000000000
           PotentialPrefactor[au]  230.
!           PotentialPrefactor[au]  10.
           PotentialPowerExponent  6
```

```
      End
     Frequencies[1/cm]            3
   1217.89 2735.01 2750.20
  ZeroEnergy[kJ/mol]            0.
  ElectronicLevels[1/cm]                   2
   0.0000000000000000        4.0000000000000000
   881.00000000000000        2.0000000000000000
 End
 !************************************
 Barrier B3  WP PRODS
   RRHO
    Stoichiometry  H2S1Cl1
    Core PhaseSpaceTheory
      FragmentGeometry[angstrom]               2
   H                  0.00000000    0.00000000   -1.26323400
   S                  0.00000000    0.00000000    0.07895200
      FragmentGeometry[angstrom]               2
   H                  0.00000000    0.00000000   -1.20561900
   Cl                 0.00000000    0.00000000    0.07091900
  SymmetryFactor     2.0000000000000000
          PotentialPrefactor[au]  230.
          PotentialPowerExponent  6
      End
    Frequencies[1/cm]            2
    2719.96 3006.51
ZeroEnergy[kJ/mol]    -53.18
 ElectronicLevels[1/cm]                   2
  0.0000000000000000        2.0000000000000000
  376.95999999999998        2.0000000000000000
End
 !************************************
 Barrier B2 WR WP
 RRHO            !
 Geometry[angstrom]            4
 Cl                 1.40626100    0.00424200   -0.01712600
 H                 -0.08527300   -0.19676000    0.61791700
 S                 -1.40388600   -0.07129300   -0.02507700
 H                 -1.35899500    1.26533600    0.07445500
  Core  RigidRotor
           SymmetryFactor   0.50000000000000000
    End
Tunneling    Eckart
    ImaginaryFrequency[1/cm]     759.25
    WellDepth[kJ/mol]     29.03
    WellDepth[kJ/mol]     52.32
    End
     Frequencies[1/cm]            5
     232.63 422.36 1013.96 1427.75 2728.71
     ZeroEnergy[kJ/mol]      -9.32
     ElectronicLevels[1/cm]                      1
     0.0000000000000000        2.0000000000000000
     End
  End
```

CBS-CVH

```
!**************************************************
!               GLOBAL SECTION
!**************************************************
!!!!!!!!!!!!!!!!!!!!!!!!!!!!!!!!!!!!!!!!!!!!!!!!!!!
!
!
TemperatureList[K]                      200 225 250 275 300 325 350 375 400 425 430 450 475 500 600 700 800 900 1000
PressureList[atm]                       0.5 1
!
!
EnergyStepOverTemperature               .2
ExcessEnergyOverTemperature             30
ModelEnergyLimit[kcal/mol]              400
!
CalculationMethod                       direct
!
WellCutoff                              20
ChemicalEigenvalueMax                   0.2
!
ReductionMethod                         diagonalization
!
AtomDistanceMin[bohr]                  1.3
!!
RateOutput                              heatlike.out
!
!
!!!!!!!!!!!!!!!!!!!!!!!!!!!!!!!!!!!!!!!!!!!!!!!!!!!
!**************************************************
!               MODEL SECTION
!**************************************************
!!!!!!!!!!!!!!!!!!!!!!!!!!!!!!!!!!!!!!!!!!!!!!!!!!!
!
!
Model
!
  EnergyRelaxation
    Exponential
       Factor[1/cm]                     260
       Power                            0.875
       ExponentCutoff                   10
    End
!
  CollisionFrequency
    LennardJones
       Epsilons[K]                       90.58  617.0
       Sigmas[angstrom]                  3.54    5.62
       Masses[amu]                       39.948 69.0
   End
!
!************************************************
!
```

```
!***************************************************
!!!!!!!!!!!!!!!!!!!!!!!!!!!!!!!!!!!!!!!!!!!!!!!!!!!!
!***************************************************
!  REACTANTS
!***************************************************


 Bimolecular REACS
 Fragment REACT1
 RRHO
 Geometry[angstrom]               3
 S                  0.00000000    0.00000000    0.10283933
 H                  0.00000000    0.96667700   -0.82271467
 H                  0.00000000   -0.96667700   -0.82271467
  Core  RigidRotor
  SymmetryFactor    2.0000000000000000
  End
     Frequencies[1/cm]            3
   1217.90 2736.90 2751.70
  ZeroEnergy[kJ/mol]            0.
  ElectronicLevels[1/cm]                      1
   0           1
 !************************************
 End
!*********
 Fragment REACT2
  Atom
  Name Cl
  ElectronicLevels[1/cm]                      2
   0       4
   881.00  2

 !************************************
 End
!*********
GroundEnergy[kJ/mol] 0.0
End
 Bimolecular PRODS
 Fragment PROD1
 RRHO
 Geometry[angstrom]               2
   H                  0.00000000    0.00000000   -1.26323400
   S                  0.00000000    0.00000000    0.07895200
  Core  RigidRotor
  SymmetryFactor    1.0000000000000000
  End
     Frequencies[1/cm]            1
   2719.97
  ZeroEnergy[kJ/mol]            0.
  ElectronicLevels[1/cm]                      2
   0.0000000000000000        2.0000000000000000
   376.95999999999998        2.0000000000000000
 !************************************
 End
!*********
 Fragment PROD2
```

```
 RRHO
 Geometry[angstrom]               2
   H                  0.00000000    0.00000000   -1.20561900
   Cl                 0.00000000    0.00000000    0.07091900
  Core  RigidRotor
  SymmetryFactor    1.0000000000000000
  End
     Frequencies[1/cm]            1
   3006.52
  ZeroEnergy[kJ/mol]            0.
  ElectronicLevels[1/cm]                     1
   0.0000000000000000        1.0000000000000000

 !************************************
 End
GroundEnergy[kJ/mol]   -51.383
End
!*********
 Well WR
 Species
 RRHO     ! well
 Geometry[angstrom]               4
   H                  0.86853200   -1.21794200    0.97069800
   S                 -0.05263800   -1.26295000    0.00000000
   H                  0.86853200   -1.21794200   -0.97069800
   Cl                -0.05263800    1.33194700    0.00000000
  Core  RigidRotor
  SymmetryFactor    1.0000000000000000
  End
     Frequencies[1/cm]            6
   235.51 361.29 394.61 1211.09 2735.17 2749.91
  ZeroEnergy[kJ/mol]     -39.68
  ElectronicLevels[1/cm]                     1
   0.0000000000000000        2.0000000000000000
 End
 End
 !************************************
 Well WP
 Species
 RRHO     ! well
 Geometry[angstrom]               4
    S                 -1.89837200   -0.07864900   -0.00000300
    H                 -1.96120000    1.26293700   -0.00000900
    H                  0.58255000   -0.07505800    0.00001500
    Cl                 1.86780000    0.00414800    0.00000200
   Core  RigidRotor
   SymmetryFactor    1.0000000000000000
  End
    Frequencies[1/cm]            6
 92.55 144.41 254.63 397.12 2715.09 2844.57
  ZeroEnergy[kJ/mol]             -61.47
  ElectronicLevels[1/cm]                     1
   0.0000000000000000        2.0000000000000000
 End
 End
 !************************************
```

```
 Barrier B1  REACS WR
   RRHO
     Stoichiometry  H2S1Cl1
     Core PhaseSpaceTheory
       FragmentGeometry[angstrom]               3
 S                  0.00000000    0.00000000    0.10283933
 H                  0.00000000    0.96667700   -0.82271467
 H                  0.00000000   -0.96667700   -0.82271467
       FragmentGeometry[angstrom]               1
 Cl                   0. 0. 0.
  SymmetryFactor     2.000000000000000
           PotentialPrefactor[au]  230.
!           PotentialPrefactor[au]  10.
           PotentialPowerExponent  6
      End
     Frequencies[1/cm]            3
   1217.89 2735.01 2750.20
  ZeroEnergy[kJ/mol]            0.
  ElectronicLevels[1/cm]                   2
   0.0000000000000000        4.0000000000000000
   881.00000000000000        2.0000000000000000
 End
 !************************************
 Barrier B3  WP PRODS
   RRHO
    Stoichiometry  H2S1Cl1
    Core PhaseSpaceTheory
      FragmentGeometry[angstrom]               2
   H                  0.00000000    0.00000000   -1.26323400
   S                  0.00000000    0.00000000    0.07895200
      FragmentGeometry[angstrom]               2
   H                  0.00000000    0.00000000   -1.20561900
   Cl                 0.00000000    0.00000000    0.07091900
  SymmetryFactor     2.0000000000000000
          PotentialPrefactor[au]  230.
          PotentialPowerExponent  6
      End
    Frequencies[1/cm]            2
    2719.96 3006.51
ZeroEnergy[kJ/mol]    -51.38
 ElectronicLevels[1/cm]                   2
  0.0000000000000000        2.0000000000000000
  376.95999999999998        2.0000000000000000
End
 !************************************
 Barrier B2 WR WP
 RRHO           !
 Geometry[angstrom]            4
 Cl                 1.40626100    0.00424200   -0.01712600
 H                 -0.08527300   -0.19676000    0.61791700
 S                 -1.40388600   -0.07129300   -0.02507700
 H                 -1.35899500    1.26533600    0.07445500
  Core  RigidRotor
           SymmetryFactor   0.50000000000000000
    End
Tunneling    Eckart
```

```
    ImaginaryFrequency[1/cm]     759.25
    WellDepth[kJ/mol]     30.37
    WellDepth[kJ/mol]     52.16
    End
     Frequencies[1/cm]            5
     232.63 422.36 1013.96 1427.75 2728.71
     ZeroEnergy[kJ/mol]      -9.31
     ElectronicLevels[1/cm]                      1
     0.0000000000000000        2.0000000000000000
     End
  End
```

CBS-QB3

```
!****************************************************
!              GLOBAL SECTION
!****************************************************
!!!!!!!!!!!!!!!!!!!!!!!!!!!!!!!!!!!!!!!!!!!!!!!!!!!!
!
!
TemperatureList[K]                      200 225 250 275 300 325 350 375 400 425 430 450 475 500 600 700 800 900 1000
PressureList[atm]                       0.5 1
!
!
EnergyStepOverTemperature               .2
ExcessEnergyOverTemperature             30
ModelEnergyLimit[kcal/mol]              400
!
CalculationMethod                       direct
!
WellCutoff                              20
ChemicalEigenvalueMax                   0.2
!
ReductionMethod                         diagonalization
!
AtomDistanceMin[bohr]                  1.3
!!
RateOutput                              qb3.out
!
!
!!!!!!!!!!!!!!!!!!!!!!!!!!!!!!!!!!!!!!!!!!!!!!!!!!!!
!****************************************************
!              MODEL SECTION
!****************************************************
!!!!!!!!!!!!!!!!!!!!!!!!!!!!!!!!!!!!!!!!!!!!!!!!!!!!
!
!
Model
!
  EnergyRelaxation
    Exponential
```

```
      Factor[1/cm]                    260
      Power                           0.875
      ExponentCutoff                  10
   End
!
  CollisionFrequency
    LennardJones
      Epsilons[K]                      90.58  617.0
      Sigmas[angstrom]                 3.54    5.62
      Masses[amu]                      39.948 69.0
   End
!
!**************************************************
!
!****************************************************
!!!!!!!!!!!!!!!!!!!!!!!!!!!!!!!!!!!!!!!!!!!!!!!!!!!!
!****************************************************
!  REACTANTS
!****************************************************


 Bimolecular REACS
 Fragment REACT1
 RRHO
 Geometry[angstrom]              3
 H                  0.00000000    0.97117900   -0.82531100
 S                  0.00000000    0.00000000    0.10316400
 H                  0.00000000   -0.97117900   -0.82531100
  Core  RigidRotor
  SymmetryFactor    2.0000000000000000
  End
     Frequencies[1/cm]           3
   1213.82 2676.31 2690.23
  ZeroEnergy[kJ/mol]            0.
  ElectronicLevels[1/cm]                      1
   0           1
 !************************************
 End
!*********
 Fragment REACT2
  Atom
  Name Cl
  ElectronicLevels[1/cm]                      2
   0       4
   881.00  2

 !************************************
 End
!*********
GroundEnergy[kJ/mol] 0.0
End
 Bimolecular PRODS
 Fragment PROD1
 RRHO
 Geometry[angstrom]              2
 H                  0.00000000    0.00000000   -1.26857400
```

```
 S                  0.00000000    0.00000000    0.07928600
  Core  RigidRotor
  SymmetryFactor    1.0000000000000000
  End
     Frequencies[1/cm]            1
   2659.89
  ZeroEnergy[kJ/mol]             0.
  ElectronicLevels[1/cm]                      2
   0.0000000000000000        2.0000000000000000
   376.95999999999998        2.0000000000000000
 !************************************
 End
!*********
 Fragment PROD2
 RRHO
 Geometry[angstrom]               2
    H                  0.00000000    0.00000000   -1.21155800
    Cl                 0.00000000    0.00000000    0.07126800
  Core  RigidRotor
  SymmetryFactor    1.0000000000000000
  End
     Frequencies[1/cm]            1
   2936.93
  ZeroEnergy[kJ/mol]             0.
  ElectronicLevels[1/cm]                      1
   0.0000000000000000        1.0000000000000000

 !************************************
 End
GroundEnergy[kJ/mol]   -59.25
End
!*********
 Well WR
 Species
 RRHO     ! well
 Geometry[angstrom]               4
 H                  0.87236400   -1.25413500    0.97382500
 S                 -0.05287100   -1.30634600    0.00000000
 H                  0.87236400   -1.25413500   -0.97382500
 Cl                -0.05287100    1.37704700    0.00000000
  Core  RigidRotor
  SymmetryFactor    1.0000000000000000
  End
     Frequencies[1/cm]            6
   215.04 338.57 371.63 1210.51 2677.85 2691.64
  ZeroEnergy[kJ/mol]     -42.68
  ElectronicLevels[1/cm]                      1
   0.0000000000000000        2.0000000000000000
 End
 End
 !************************************
 Well WP
 Species
 RRHO     ! well
 Geometry[angstrom]               4
 S                  1.88505200   -0.08051500    0.00020300
```

```
 H                  2.01586800    1.26180900    0.00006400
 H                 -0.56242200   -0.05095100    0.00032000
 Cl                -1.85966300    0.00455200   -0.00021400
   Core  RigidRotor
   SymmetryFactor    1.0000000000000000
  End
    Frequencies[1/cm]            6
 93.81 170.86 281.81 435.99 2656.40 2710.28
  ZeroEnergy[kJ/mol]              -62.50
  ElectronicLevels[1/cm]                      1
   0.0000000000000000        2.0000000000000000
 End
 End
 !************************************
 Barrier B1  REACS WR
   RRHO
     Stoichiometry  H2S1Cl1
     Core PhaseSpaceTheory
       FragmentGeometry[angstrom]               3
 H                  0.00000000    0.97117900   -0.82531100
 S                  0.00000000    0.00000000    0.10316400
 H                  0.00000000   -0.97117900   -0.82531100
       FragmentGeometry[angstrom]               1
 Cl                   0. 0. 0.
  SymmetryFactor     2.000000000000000
           PotentialPrefactor[au]  230.
!           PotentialPrefactor[au]  10.
           PotentialPowerExponent  6
      End
     Frequencies[1/cm]            3
   1213.82 2676.31 2690.23
  ZeroEnergy[kJ/mol]            0.
  ElectronicLevels[1/cm]                   2
   0.0000000000000000        4.0000000000000000
   881.00000000000000        2.0000000000000000
 End
 !************************************
 Barrier B3  WP PRODS
   RRHO
    Stoichiometry  H2S1Cl1
    Core PhaseSpaceTheory
      FragmentGeometry[angstrom]               2
   H                  0.00000000    0.00000000   -1.26857400
   S                  0.00000000    0.00000000    0.07928600
      FragmentGeometry[angstrom]               2
   H                  0.00000000    0.00000000   -1.21155800
   Cl                 0.00000000    0.00000000    0.07126800
  SymmetryFactor     2.0000000000000000
          PotentialPrefactor[au]  230.
          PotentialPowerExponent  6
      End
    Frequencies[1/cm]            2
     2659.89 2936.93
ZeroEnergy[kJ/mol]    -59.25
 ElectronicLevels[1/cm]                   2
  0.0000000000000000        2.0000000000000000
```

```
  376.95999999999998        2.0000000000000000
End
 !************************************
 Barrier B2 WR WP
 RRHO            !
 Geometry[angstrom]            4
    Cl                 1.46141200    0.00420700   -0.01507200
 H                 -0.04359800   -0.14801900    0.52866300
 S                 -1.45887600   -0.07462900   -0.01976900
 H                 -1.45838600    1.27056300    0.04385800
  Core  RigidRotor
           SymmetryFactor   0.50000000000000000
    End
Tunneling    Eckart
    ImaginaryFrequency[1/cm]     1016.86
    WellDepth[kJ/mol]     27.77
    WellDepth[kJ/mol]     47.58
    End
     Frequencies[1/cm]            5
     211.63 409.67 979.18 1162.65 2669.09
     ZeroEnergy[kJ/mol]      -14.92
     ElectronicLevels[1/cm]                      1
     0.0000000000000000        2.0000000000000000
     End
  End
```

$CH_3NH_2 + CN^{\bullet}$

CN attack to the CH_3 moiety

jChS

```
!***************************************************
!              GLOBAL SECTION
!***************************************************
!!!!!!!!!!!!!!!!!!!!!!!!!!!!!!!!!!!!!!!!!!!!!!!!!!!!
!
!
TemperatureList[K]                     30 40 50 60 70 80 90 100 110 120 130 140 150 160 170 180 190 200 220 240 260 280 300 350 400 450 500
PressureList[atm]                       1.e-8
!
!
EnergyStepOverTemperature               .2
ExcessEnergyOverTemperature             30
ModelEnergyLimit[kcal/mol]              400
!
CalculationMethod                        low-eigenvalue
!
WellCutoff                              20
```

```
ChemicalEigenvalueMax                   0.2
!
ReductionMethod                         diagonalization
AtomDistanceMin[bohr]                  1.3
!!
RateOutput                              fc01_ChS.out
!
!
!!!!!!!!!!!!!!!!!!!!!!!!!!!!!!!!!!!!!!!!!!!!!!!!!!!!
!***************************************************
!               MODEL SECTION
!***************************************************
!!!!!!!!!!!!!!!!!!!!!!!!!!!!!!!!!!!!!!!!!!!!!!!!!!!!
!
!
Model
!
  EnergyRelaxation
    Exponential
       Factor[1/cm]                     260
       Power                            0.875
       ExponentCutoff                   10
    End
!
   CollisionFrequency
    LennardJones
       Epsilons[K]                       127.697   1382.375
       Sigmas[angstrom]                    3.462   4.046
       Masses[amu]                       39.948 70.07
   End
!
!*************************************************
!
!***************************************************
!!!!!!!!!!!!!!!!!!!!!!!!!!!!!!!!!!!!!!!!!!!!!!!!!!!!
!***************************************************
!  REACTANTS  - CH3NH2 + CN
!***************************************************
 Bimolecular REACS
 Fragment CH3NH2
 RRHO
 Geometry[angstrom]               7
 C                 -0.74064000    0.00000000    0.01360800
 H                 -1.12725000   -0.87638300   -0.50253800
 H                 -1.14607300    0.00000000    1.03050000
 H                 -1.12725000    0.87638400   -0.50253700
 N                  0.72026000    0.00000000   -0.07006200
 H                  1.10585300    0.81199300    0.39300800
 H                  1.10585300   -0.81199300    0.39300800
  Core  RigidRotor
  SymmetryFactor    1.0000000000000000
  End
     Frequencies[1/cm]         15
     300.20
     834.61
     978.18
```

```
     1064.10
     1178.30
     1354.60
     1472.48
     1517.20
     1536.05
     1672.27
     3006.41
     3092.76
     3129.63
     3519.95
     3601.33
  ZeroEnergy[kJ/mol]            0.
  ElectronicLevels[1/cm]                     1
   0.0000000000000000       1.0000000000000000
 !************************************
 End
!*********
 Fragment CN
 RRHO
 Geometry[angstrom]              2
 C                 0.00000000    0.00000000   -0.63181100
 N                 0.00000000    0.00000000    0.54143400
  Core  RigidRotor
  SymmetryFactor    1.0000000000000000
  End
     Frequencies[1/cm]           1
    2076.80
  ZeroEnergy[kJ/mol]            0.
  ElectronicLevels[1/cm]                     1
   0.0000000000000000       2.0000000000000000
  !************************************
 End
!*********
GroundEnergy[kJ/mol] 0.0
End
!****************************************************
!  PRODUCT P1  - CH2NH2 + HCN
!****************************************************
 Bimolecular P1
 Fragment CH2NH2
 RRHO
 Geometry[angstrom]              6
  C                 0.73716300    0.05207300    0.00000000
 H                 1.24098000   -0.16233200    0.92778800
 H                 1.24098000   -0.16233200   -0.92778800
 N                -0.65004100   -0.05946900    0.00000000
 H                -1.11367100    0.26546000    0.83278100
 H                -1.11367100    0.26546000   -0.83278100
  Core  RigidRotor
  SymmetryFactor    1.0000000000000000
  End
     Frequencies[1/cm]           12
455.87
608.48
672.66
```

```
941.68
1228.92
1332.81
1494.17
1661.62
3175.68
3289.29
3563.85
3668.18
  ZeroEnergy[kJ/mol]            0.
  ElectronicLevels[1/cm]                      1
   0.0000000000000000        2.0000000000000000
    !************************************
 End
!*********
 Fragment HCN
 RRHO
 Geometry[angstrom]               3
  C                  0.00000000    0.00000000   -0.55868200
  H                  0.00000000    0.00000000   -1.62356200
  N                  0.00000000    0.00000000    0.59561500
  Core  RigidRotor
  SymmetryFactor    1.0000000000000000
  End
     Frequencies[1/cm]            4
    747.43
    747.43
    2124.86
    3444.80
  ZeroEnergy[kJ/mol]            0.
  ElectronicLevels[1/cm]                      1
   0.0000000000000000        1.0000000000000000
  !************************************
 End
!*********
GroundEnergy[kJ/mol] -141.40
End
!***************************************************
!  PRODUCT P2  - AAN + H
!***************************************************
Bimolecular P2
 Fragment AAN
 RRHO
 Geometry[angstrom]               8
  N                 -1.57103400   -0.47328200   -0.06614300
  H                 -1.48750500   -1.09302700    0.72899000
  H                 -2.51570100   -0.11296900   -0.07859300
  C                 -0.61799900    0.62930100    0.03134800
  H                 -0.75621900    1.28659100   -0.82625400
  H                 -0.71181000    1.24606600    0.93131000
  C                  0.75714200    0.12420400   -0.00760700
  N                  1.84557000   -0.26792000   -0.00857200
  Core  RigidRotor
  SymmetryFactor    1.0000000000000000
  End
     Frequencies[1/cm]            18
```

```
200.35
247.71
372.97
569.46
821.26
878.29
1011.17
1090.52
1198.39
1316.21
1430.00
1511.75
1668.36
2294.29
3014.18
3099.75
3522.30
3610.24
  ZeroEnergy[kJ/mol]            0.
  ElectronicLevels[1/cm]                     1
   0.0000000000000000       1.0000000000000000
    !************************************
 End
!*********
 Fragment HYDROGEN
  Atom
  Name H
  ElectronicLevels[1/cm]                     1
   0.0000000000000000       2.0000000000000000
    !************************************
 End
GroundEnergy[kJ/mol]   -84.10
End

Well FC01
 Species
 RRHO    ! transition state
 Geometry[angstrom]              9
 C                -1.75961900    0.70751200    0.00000200
 N                -1.94559400   -0.66454800    0.00000100
 H                -1.96563500    1.22008800   -0.92616900
 H                -1.96559300    1.22008100    0.92618500
 H                 0.53087300    0.59788100   -0.00001100
 H                -1.70666100   -1.16502300   -0.83939700
 H                -1.70662200   -1.16502800    0.83938500
 C                 1.54268600    0.22600200   -0.00000600
 N                 2.62188500   -0.18638800    0.00000300
  Core  RigidRotor
  SymmetryFactor    1.000000000000000
  End
     Frequencies[1/cm]         21
     40.34 71.38 136.95 149.68 185.17
     511.30 575.14 729.22 848.06 861.82
     943.55 1246.23 1328.18 1493.79 1665.76
     2104.04 3165.10 3238.70 3277.59 3579.46
     3687.37
```

```
     ZeroEnergy[kJ/mol]               -155.30
  ElectronicLevels[1/cm]                      1
   0.0000000000000000        2.0000000000000000
 End
 End
!*********
Well RI
 Species
 RRHO     ! transition state
    Geometry[angstrom]               9
  C                -0.81668500   -0.17355500    0.35900500
  N                -1.83377000   -0.09517100   -0.34924000
  C                 0.47780400    0.59647500    0.08989800
  H                 0.69617500    1.20943200    0.96394100
  N                 1.62364600   -0.25534900   -0.18366100
  H                 1.83966100   -0.85986400    0.59799600
  H                 0.31432000    1.26303800   -0.75239800
  H                 1.45881200   -0.84041500   -0.99214800
  H                -0.80481900   -0.85607000    1.21949000
    Core  RigidRotor
     SymmetryFactor    1.000000000000000
    End
     Frequencies[1/cm]            21
     111.64
     295.78
     361.91
     429.13
     781.88
     814.81
     917.98
     961.28
     1098.84
     1131.21
     1254.02
     1353.86
     1386.79
     1495.95
     1670.85
     1726.45
     2985.23
     3076.44
     3139.85
     3519.41
     3606.32
     ZeroEnergy[kJ/mol]         -177.40
     ElectronicLevels[1/cm]                      1
     0.0000000000000000        2.0000000000000000
 End
 End
Barrier L1  REACS FC01
   RRHO
    Stoichiometry  C2N2H5
     Core PhaseSpaceTheory
            FragmentGeometry[angstrom]               7
        C                -0.74064000    0.00000000    0.01360800
        H                -1.12725000   -0.87638300   -0.50253800
```

```
        H                 -1.14607300    0.00000000    1.03050000
        H                 -1.12725000    0.87638400   -0.50253700
        N                  0.72026000    0.00000000   -0.07006200
        H                  1.10585300    0.81199300    0.39300800
        H                  1.10585300   -0.81199300    0.39300800
            FragmentGeometry[angstrom]               2
            C                  0.00000000    0.00000000   -0.63181100
            N                  0.00000000    0.00000000    0.54143400
       SymmetryFactor     1.0000000000000000
              PotentialPrefactor[au]        64.2
           PotentialPowerExponent  6
  End
Frequencies[1/cm]     16
      300.20
      834.61
      978.18
      1064.10
1178.30
1354.60
1472.48
1517.20
1536.05
1672.27
2076.80
3006.41
3092.76
3129.63
3519.95
3601.33
  ZeroEnergy[kJ/mol]            0.00
  ElectronicLevels[1/cm]                   1
   0.0000000000000000        2.0000000000000000
   End
Barrier TS0 FC01 RI
  RRHO
   Geometry[angstrom]            9
 C                  0.00000000   -1.09720900    0.00000000
 N                  1.35452600   -0.84778800    0.00000000
 C                 -1.11626800    0.70603600    0.00000000
 N                 -0.47146700    1.69048500    0.00000000
 H                 -0.39627600   -1.51179700   -0.91690600
 H                 -0.39627600   -1.51179700    0.91690600
 H                 -2.09283800    0.25152700    0.00000000
 H                  1.70079300   -0.38988800   -0.82932000
 H                  1.70079300   -0.38988800    0.82932000
  Core  RigidRotor
    SymmetryFactor   1.0
  End
  Tunneling    Eckart
    ImaginaryFrequency[1/cm] 439.55
    WellDepth[kJ/mol]     43.6
    WellDepth[kJ/mol]     65.7
  End
  Frequencies[1/cm]            20
  68.52 174.51 303.62 417.15 639.48
  733.00 765.24 810.83 989.56 1002.28
```

```
  1250.86 1360.71 1504.14 1679.45 1971.74
  3132.64 3236.08 3238.57 3555.29 3650.56
  ZeroEnergy[kJ/mol]  -111.70
  ElectronicLevels[1/cm]                    1
      0        2
 End
 !************************************
Barrier TS1 RI P1
  RRHO
   Geometry[angstrom]           9
  C                 -0.37637500   -1.15785300    0.00000000
 N                 -1.50393100   -1.48970200    0.00000000
 C                  0.00000000    0.94119800    0.00000000
 H                 -0.52635600    1.16094400    0.91597600
 N                  1.34770700    1.24412200    0.00000000
 H                  1.86810200    1.06171100    0.84146500
 H                 -0.52635600    1.16094400   -0.91597600
 H                  1.86810200    1.06171100   -0.84146500
 H                  0.66832000   -1.42632400    0.00000000
  Core  RigidRotor
    SymmetryFactor   1.0
  End
  Tunneling    Eckart
    ImaginaryFrequency[1/cm]   410.85
    WellDepth[kJ/mol]          69.3
    WellDepth[kJ/mol]          33.3
  End
   Frequencies[1/cm]            20
   47.12 198.55   350.87   369.37   533.13
   629.46   765.47   840.02   981.05   983.13
   1231.41   1337.86   1496.14   1669.51   1977.54
   3160.36   3212.85   3268.30   3580.86   3689.26
  ZeroEnergy[kJ/mol]  -108.10
    ElectronicLevels[1/cm]                    1
      0        2
 End
 !************************************
Barrier TS2 RI P2
  RRHO
   Geometry[angstrom]           9
 C                 -0.73927000   -0.02404700    0.00004600
 N                 -1.85483100   -0.37639800    0.00001400
 C                  0.59330900    0.60138700    0.00002100
 H                  0.65490000    1.24835300    0.87485500
 N                  1.73184700   -0.30796900   -0.00002000
 H                  1.71082400   -0.90752400    0.81447000
 H                 -2.99472100    0.64481000   -0.00036300
 H                  0.65486100    1.24837800   -0.87480000
 H                  1.71079000   -0.90749300   -0.81453100
  Core  RigidRotor
    SymmetryFactor   1.0
  End
  Tunneling    Eckart
    ImaginaryFrequency[1/cm]   1219.30
    WellDepth[kJ/mol]          120.1
    WellDepth[kJ/mol]          26.8
```

```
    End
   Frequencies[1/cm]            20
  10.91
  182.81
  263.95
  415.59
  441.95
  649.53
  825.77
  890.51
  918.53
  1105.48
  1194.16
  1365.77
  1391.15
  1474.75
  1676.62
  2189.59
  3070.93
  3113.30
  3522.03
  3605.56
  ZeroEnergy[kJ/mol]  -57.30
    ElectronicLevels[1/cm]                      1
       0        2
 End
End
```

CBS-CVH

```
!***************************************************
!               GLOBAL SECTION
!***************************************************
!!!!!!!!!!!!!!!!!!!!!!!!!!!!!!!!!!!!!!!!!!!!!!!!!!!!
!
!
TemperatureList[K]                     30 40 50 60 70 80 90 100 110 120 130 140 150 160 170 180 190 200 220 240 260 280 300 350 400 450 500
PressureList[atm]                       1.e-8
!
!
EnergyStepOverTemperature               .2
ExcessEnergyOverTemperature             30
ModelEnergyLimit[kcal/mol]              400
!
CalculationMethod                        low-eigenvalue
!
WellCutoff                              20
ChemicalEigenvalueMax                   0.2
!
ReductionMethod                         diagonalization
!
AtomDistanceMin[bohr]                  1.3
!!
RateOutput                              fc01_HEATlike.out
!
!
```

```
!!!!!!!!!!!!!!!!!!!!!!!!!!!!!!!!!!!!!!!!!!!!!!!!!!!!
!***************************************************
!               MODEL SECTION
!***************************************************
!!!!!!!!!!!!!!!!!!!!!!!!!!!!!!!!!!!!!!!!!!!!!!!!!!!!
!
!
Model
!
  EnergyRelaxation
    Exponential
       Factor[1/cm]                     260
       Power                            0.875
       ExponentCutoff                   10
    End
!
   CollisionFrequency
    LennardJones
       Epsilons[K]                       127.697   1382.375
       Sigmas[angstrom]                    3.462   4.046
       Masses[amu]                       39.948 70.07
   End
!
!*************************************************
!
!***************************************************
!!!!!!!!!!!!!!!!!!!!!!!!!!!!!!!!!!!!!!!!!!!!!!!!!!!!
!***************************************************
!  REACTANTS  - CH3NH2 + CN
!***************************************************
 Bimolecular REACS
 Fragment CH3NH2
 RRHO
 Geometry[angstrom]               7
 C                 -0.74064000    0.00000000    0.01360800
 H                 -1.12725000   -0.87638300   -0.50253800
 H                 -1.14607300    0.00000000    1.03050000
 H                 -1.12725000    0.87638400   -0.50253700
 N                  0.72026000    0.00000000   -0.07006200
 H                  1.10585300    0.81199300    0.39300800
 H                  1.10585300   -0.81199300    0.39300800
  Core  RigidRotor
  SymmetryFactor    1.0000000000000000
  End
     Frequencies[1/cm]         15
     300.20
     834.61
     978.18
     1064.10
     1178.30
     1354.60
     1472.48
     1517.20
     1536.05
     1672.27
     3006.41
```

```
    3092.76
    3129.63
    3519.95
    3601.33
  ZeroEnergy[kJ/mol]            0.
  ElectronicLevels[1/cm]                     1
   0.0000000000000000       1.0000000000000000
 !************************************
 End
!*********
 Fragment CN
 RRHO
 Geometry[angstrom]              2
 C                  0.00000000    0.00000000   -0.63181100
 N                  0.00000000    0.00000000    0.54143400
  Core  RigidRotor
  SymmetryFactor    1.0000000000000000
  End
    Frequencies[1/cm]           1
   2076.80
  ZeroEnergy[kJ/mol]            0.
  ElectronicLevels[1/cm]                     1
   0.0000000000000000       2.0000000000000000
  !************************************
 End
!*********
GroundEnergy[kJ/mol] 0.0
End
!****************************************************
!  PRODUCT P1  - CH2NH2 + HCN
!****************************************************
 Bimolecular P1
 Fragment CH2NH2
 RRHO
 Geometry[angstrom]              6
  C                  0.73716300    0.05207300    0.00000000
 H                  1.24098000   -0.16233200    0.92778800
 H                  1.24098000   -0.16233200   -0.92778800
 N                 -0.65004100   -0.05946900    0.00000000
 H                 -1.11367100    0.26546000    0.83278100
 H                 -1.11367100    0.26546000   -0.83278100
  Core  RigidRotor
  SymmetryFactor    1.0000000000000000
  End
    Frequencies[1/cm]           12
455.87
608.48
672.66
941.68
1228.92
1332.81
1494.17
1661.62
3175.68
3289.29
3563.85
```

```
3668.18
  ZeroEnergy[kJ/mol]            0.
  ElectronicLevels[1/cm]                    1
   0.0000000000000000       2.0000000000000000
    !************************************
 End
!*********
 Fragment HCN
 RRHO
 Geometry[angstrom]              3
  C                 0.00000000    0.00000000   -0.55868200
  H                 0.00000000    0.00000000   -1.62356200
  N                 0.00000000    0.00000000    0.59561500
  Core  RigidRotor
  SymmetryFactor    1.0000000000000000
  End
     Frequencies[1/cm]           4
    747.43
    747.43
    2124.86
    3444.80
  ZeroEnergy[kJ/mol]            0.
  ElectronicLevels[1/cm]                    1
   0.0000000000000000       1.0000000000000000
  !************************************
 End
!*********
GroundEnergy[kJ/mol] -140.20
End
!****************************************************
!  PRODUCT P2  - AAN + H
!****************************************************
Bimolecular P2
 Fragment AAN
 RRHO
 Geometry[angstrom]              8
  N                -1.57103400   -0.47328200   -0.06614300
  H                -1.48750500   -1.09302700    0.72899000
  H                -2.51570100   -0.11296900   -0.07859300
  C                -0.61799900    0.62930100    0.03134800
  H                -0.75621900    1.28659100   -0.82625400
  H                -0.71181000    1.24606600    0.93131000
  C                 0.75714200    0.12420400   -0.00760700
  N                 1.84557000   -0.26792000   -0.00857200
  Core  RigidRotor
  SymmetryFactor    1.0000000000000000
  End
     Frequencies[1/cm]           18
200.35
247.71
372.97
569.46
821.26
878.29
1011.17
1090.52
```

```
1198.39
1316.21
1430.00
1511.75
1668.36
2294.29
3014.18
3099.75
3522.30
3610.24
  ZeroEnergy[kJ/mol]            0.
  ElectronicLevels[1/cm]                     1
   0.0000000000000000       1.0000000000000000
    !************************************
 End
!*********
 Fragment HYDROGEN
  Atom
  Name H
  ElectronicLevels[1/cm]                     1
   0.0000000000000000       2.0000000000000000
    !************************************
 End
GroundEnergy[kJ/mol]   -82.80
End
Well FC01
 Species
 RRHO     ! transition state
 Geometry[angstrom]              9
 C                -1.75961900    0.70751200    0.00000200
 N                -1.94559400   -0.66454800    0.00000100
 H                -1.96563500    1.22008800   -0.92616900
 H                -1.96559300    1.22008100    0.92618500
 H                 0.53087300    0.59788100   -0.00001100
 H                -1.70666100   -1.16502300   -0.83939700
 H                -1.70662200   -1.16502800    0.83938500
 C                 1.54268600    0.22600200   -0.00000600
 N                 2.62188500   -0.18638800    0.00000300
  Core  RigidRotor
  SymmetryFactor    1.000000000000000
  End
     Frequencies[1/cm]          21
     40.34 71.38 136.95 149.68 185.17
     511.30 575.14 729.22 848.06 861.82
     943.55 1246.23 1328.18 1493.79 1665.76
     2104.04 3165.10 3238.70 3277.59 3579.46
     3687.37
     ZeroEnergy[kJ/mol]              -153.50
  ElectronicLevels[1/cm]                     1
   0.0000000000000000       2.0000000000000000
 End
 End
!*********
Well RI
 Species
 RRHO     ! transition state
```

```
    Geometry[angstrom]              9
  C                -0.81668500   -0.17355500    0.35900500
  N                -1.83377000   -0.09517100   -0.34924000
  C                 0.47780400    0.59647500    0.08989800
  H                 0.69617500    1.20943200    0.96394100
  N                 1.62364600   -0.25534900   -0.18366100
  H                 1.83966100   -0.85986400    0.59799600
  H                 0.31432000    1.26303800   -0.75239800
  H                 1.45881200   -0.84041500   -0.99214800
  H                -0.80481900   -0.85607000    1.21949000
    Core  RigidRotor
     SymmetryFactor    1.000000000000000
    End
     Frequencies[1/cm]           21
     111.64
     295.78
     361.91
     429.13
     781.88
     814.81
     917.98
     961.28
     1098.84
     1131.21
     1254.02
     1353.86
     1386.79
     1495.95
     1670.85
     1726.45
     2985.23
     3076.44
     3139.85
     3519.41
     3606.32
     ZeroEnergy[kJ/mol]         -176.90
     ElectronicLevels[1/cm]                     1
     0.0000000000000000        2.0000000000000000
 End
 End
!!*************************************
Barrier L1  REACS FC01
   RRHO
    Stoichiometry  C2N2H5
     Core PhaseSpaceTheory
            FragmentGeometry[angstrom]              7
        C                -0.74064000    0.00000000    0.01360800
        H                -1.12725000   -0.87638300   -0.50253800
        H                -1.14607300    0.00000000    1.03050000
        H                -1.12725000    0.87638400   -0.50253700
        N                 0.72026000    0.00000000   -0.07006200
        H                 1.10585300    0.81199300    0.39300800
        H                 1.10585300   -0.81199300    0.39300800
            FragmentGeometry[angstrom]              2
            C                 0.00000000    0.00000000   -0.63181100
            N                 0.00000000    0.00000000    0.54143400
```

```
      SymmetryFactor     1.0000000000000000
             PotentialPrefactor[au]        64.2
          PotentialPowerExponent  6
  End
Frequencies[1/cm]     16
      300.20
      834.61
      978.18
      1064.10
1178.30
1354.60
1472.48
1517.20
1536.05
1672.27
2076.80
3006.41
3092.76
3129.63
3519.95
3601.33
  ZeroEnergy[kJ/mol]            0.00
  ElectronicLevels[1/cm]                  1
   0.0000000000000000        2.0000000000000000
   End
!*********************************
Barrier TS0 FC01 RI
  RRHO
   Geometry[angstrom]            9
 C                 0.00000000   -1.09720900    0.00000000
 N                 1.35452600   -0.84778800    0.00000000
 C                -1.11626800    0.70603600    0.00000000
 N                -0.47146700    1.69048500    0.00000000
 H                -0.39627600   -1.51179700   -0.91690600
 H                -0.39627600   -1.51179700    0.91690600
 H                -2.09283800    0.25152700    0.00000000
 H                 1.70079300   -0.38988800   -0.82932000
 H                 1.70079300   -0.38988800    0.82932000
  Core  RigidRotor
    SymmetryFactor   1.0
  End
  Tunneling    Eckart
    ImaginaryFrequency[1/cm] 439.55
    WellDepth[kJ/mol]     41.6
    WellDepth[kJ/mol]     65.0
  End
  Frequencies[1/cm]            20
  68.52 174.51 303.62 417.15 639.48
  733.00 765.24 810.83 989.56 1002.28
  1250.86 1360.71 1504.14 1679.45 1971.74
  3132.64 3236.08 3238.57 3555.29 3650.56
  ZeroEnergy[kJ/mol]  -111.90
  ElectronicLevels[1/cm]                     1
      0        2
 End
 !*************************************
```

```
Barrier TS1 RI P1
  RRHO
   Geometry[angstrom]           9
  C                 -0.37637500   -1.15785300    0.00000000
 N                 -1.50393100   -1.48970200    0.00000000
 C                  0.00000000    0.94119800    0.00000000
 H                 -0.52635600    1.16094400    0.91597600
 N                  1.34770700    1.24412200    0.00000000
 H                  1.86810200    1.06171100    0.84146500
 H                 -0.52635600    1.16094400   -0.91597600
 H                  1.86810200    1.06171100   -0.84146500
 H                  0.66832000   -1.42632400    0.00000000
  Core  RigidRotor
    SymmetryFactor   1.0
  End
  Tunneling    Eckart
    ImaginaryFrequency[1/cm]   410.85
    WellDepth[kJ/mol]          68.6
    WellDepth[kJ/mol]          31.9
  End
   Frequencies[1/cm]           20
   47.12 198.55   350.87   369.37   533.13
   629.46   765.47   840.02   981.05   983.13
   1231.41   1337.86   1496.14   1669.51   1977.54
   3160.36   3212.85   3268.30   3580.86   3689.26
  ZeroEnergy[kJ/mol]  -108.10
    ElectronicLevels[1/cm]                      1
       0        2
 End
 !*************************************
Barrier TS2 RI P2
  RRHO
   Geometry[angstrom]           9
 C                 -0.73927000   -0.02404700    0.00004600
 N                 -1.85483100   -0.37639800    0.00001400
 C                  0.59330900    0.60138700    0.00002100
 H                  0.65490000    1.24835300    0.87485500
 N                  1.73184700   -0.30796900   -0.00002000
 H                  1.71082400   -0.90752400    0.81447000
 H                 -2.99472100    0.64481000   -0.00036300
 H                  0.65486100    1.24837800   -0.87480000
 H                  1.71079000   -0.90749300   -0.81453100
  Core  RigidRotor
    SymmetryFactor   1.0
  End
  Tunneling    Eckart
    ImaginaryFrequency[1/cm]   1219.30
    WellDepth[kJ/mol]          120.10
    WellDepth[kJ/mol]          26.00
    End
   Frequencies[1/cm]           20
  10.91
  182.81
  263.95
  415.59
  441.95
```

```
  649.53
  825.77
  890.51
  918.53
  1105.48
  1194.16
  1365.77
  1391.15
  1474.75
  1676.62
  2189.59
  3070.93
  3113.30
  3522.03
  3605.56
  ZeroEnergy[kJ/mol]  -56.80
    ElectronicLevels[1/cm]                      1
       0        2
 End
End
```

CBS-QB3

```
!***************************************************
!               GLOBAL SECTION
!***************************************************
!!!!!!!!!!!!!!!!!!!!!!!!!!!!!!!!!!!!!!!!!!!!!!!!!!!!
!
!
TemperatureList[K]                     30 40 50 60 70 80 90 100 110 120 130 140 150 160 170 180 190 200 220 240 260 280 300 350 400 450 500
PressureList[atm]                       1.e-8
!
!
EnergyStepOverTemperature               .2
ExcessEnergyOverTemperature             30
ModelEnergyLimit[kcal/mol]              400
!
CalculationMethod                        low-eigenvalue
!
WellCutoff                              20
ChemicalEigenvalueMax                   0.2
!
ReductionMethod                         diagonalization
AtomDistanceMin[bohr]                  1.3
!!
RateOutput                              fc01_CBSQB3.out
!
!
!!!!!!!!!!!!!!!!!!!!!!!!!!!!!!!!!!!!!!!!!!!!!!!!!!!!
!***************************************************
!               MODEL SECTION
!***************************************************
!!!!!!!!!!!!!!!!!!!!!!!!!!!!!!!!!!!!!!!!!!!!!!!!!!!!
!
!
Model
```

```
!
  EnergyRelaxation
    Exponential
       Factor[1/cm]                    260
       Power                           0.875
       ExponentCutoff                  10
    End
!
   CollisionFrequency
    LennardJones
       Epsilons[K]                      127.697   1382.375
       Sigmas[angstrom]                   3.462   4.046
       Masses[amu]                      39.948 70.07
   End
!
!**************************************************
!
!****************************************************
!!!!!!!!!!!!!!!!!!!!!!!!!!!!!!!!!!!!!!!!!!!!!!!!!!!!
!****************************************************
!  REACTANTS  - CH3NH2 + CN
!****************************************************
 Bimolecular REACS
 Fragment CH3NH2
 RRHO
 Geometry[angstrom]               7
  C                  0.05052800    0.70535200    0.00000000
 H                  0.59223700    1.06563400   -0.87850300
 H                 -0.94333700    1.18056700    0.00000000
 H                  0.59223700    1.06563400    0.87850300
 N                  0.05052800   -0.76080300    0.00000000
 H                 -0.44900100   -1.10916200    0.81210100
 H                 -0.44900100   -1.10916200   -0.81210100
  Core  RigidRotor
  SymmetryFactor    1.0000000000000000
  End
     Frequencies[1/cm]         15
316.27
851.39
979.67
1056.00
1170.62
1352.24
1461.12
1496.80
1518.88
1668.86
2947.98
3051.48
3086.76
3488.87
3562.98
  ZeroEnergy[kJ/mol]             0.
  ElectronicLevels[1/cm]                       1
   0.0000000000000000        1.0000000000000000
 !************************************
```

```
 End
!*********
 Fragment CN
 RRHO
 Geometry[angstrom]              2
 C                 0.00000000    0.00000000   -0.62766800
 N                 0.00000000    0.00000000    0.53800100
  Core  RigidRotor
  SymmetryFactor    1.0000000000000000
  End
     Frequencies[1/cm]           1
    2152.08
  ZeroEnergy[kJ/mol]            0.
  ElectronicLevels[1/cm]                     1
   0.0000000000000000       2.0000000000000000
  !************************************
 End
!*********
GroundEnergy[kJ/mol] 0.0
End
!***************************************************
!  PRODUCT P1  - CH2NH2 + HCN
!***************************************************
 Bimolecular P1
 Fragment CH2NH2
 RRHO
 Geometry[angstrom]              6
 C                 0.01156800    0.73376100    0.00000000
 H                 0.29512000    1.21317300    0.92862900
 H                 0.29512000    1.21317300   -0.92862900
 N                 0.01156800   -0.66370000    0.00000000
 H                -0.37031000   -1.09150700    0.83280600
 H                -0.37031000   -1.09150700   -0.83280600
  Core  RigidRotor
  SymmetryFactor    1.0000000000000000
  End
     Frequencies[1/cm]           12
456.57
634.33
718.25
945.40
1221.52
1327.71
1477.69
1655.79
3127.77
3235.58
3530.33
3629.03
  ZeroEnergy[kJ/mol]            0.
  ElectronicLevels[1/cm]                     1
   0.0000000000000000       2.0000000000000000
    !************************************
 End
!*********
 Fragment HCN
```

```
 RRHO
 Geometry[angstrom]               3
 C                  0.00000000    0.00000000   -0.49814100
 H                  0.00000000    0.00000000   -1.56440500
 N                  0.00000000    0.00000000    0.65046400
  Core  RigidRotor
  SymmetryFactor    1.0000000000000000
  End
     Frequencies[1/cm]            4
785.56
785.56
2200.47
3457.47
  ZeroEnergy[kJ/mol]             0.
  ElectronicLevels[1/cm]                       1
   0.0000000000000000        1.0000000000000000
  !************************************
 End
!*********
GroundEnergy[kJ/mol] -140.86
End
!***************************************************
!  PRODUCT P2  - AAN + H
!***************************************************
Bimolecular P2
 Fragment AAN
 RRHO
 Geometry[angstrom]               8
  N                 -1.48498600   -0.48890600   -0.11390600
 H                 -1.45790900   -1.10714600    0.69034800
 H                 -2.42302700   -0.10779600   -0.17350900
 C                 -0.52292900    0.60257600    0.04826100
 H                 -0.64995500    1.30621000   -0.77974700
 H                 -0.61951800    1.18427500    0.97839000
 C                  0.85402500    0.09840800   -0.00847600
 N                  1.93696200   -0.29415800   -0.02240700
  Core  RigidRotor
  SymmetryFactor    1.0000000000000000
  End
     Frequencies[1/cm]            18
208.33
247.34
384.37
575.08
822.17
875.77
999.57
1082.24
1195.93
1313.31
1421.78
1492.19
1664.62
2365.29
2959.46
3061.83
```

```
3502.01
3583.99
  ZeroEnergy[kJ/mol]            0.
  ElectronicLevels[1/cm]                     1
   0.0000000000000000        1.0000000000000000
    !************************************
 End
!*********
 Fragment HYDROGEN
  Atom
  Name H
  ElectronicLevels[1/cm]                     1
   0.0000000000000000        2.0000000000000000
    !************************************
 End
GroundEnergy[kJ/mol]   -85.55
End
!****************************
Well FC01
 Species
 RRHO     ! transition state
 Geometry[angstrom]              9
 C                 -1.55252900    0.68848400    0.00000700
 N                 -2.15266700   -0.56470500    0.00001000
 H                 -1.62056800    1.24692800   -0.92650100
 H                 -1.62057600    1.24693700    0.92650900
 H                  0.73366900    0.35009000    0.00000200
 H                 -2.05433600   -1.11687100   -0.83979300
 H                 -2.05434500   -1.11686300    0.83982000
 C                  1.78462000    0.09203600   -0.00000600
 N                  2.89889700   -0.19148700   -0.00001600
  Core  RigidRotor
  SymmetryFactor    1.000000000000000
  End
     Frequencies[1/cm]          21
27.76
86.45
117.58
157.89
192.92
518.78
616.91
775.47
890.37
902.06
950.71
1239.85
1323.73
1479.00
1659.86
2170.61
3119.15
3211.56
3225.90
3553.56
3655.27
```

```
     ZeroEnergy[kJ/mol]              -153.30
  ElectronicLevels[1/cm]                     1
   0.0000000000000000        2.0000000000000000
 End
 End
!*********
Well RI
 Species
 RRHO     ! transition state
    Geometry[angstrom]              9
 C                 -0.82498100   -0.17560100    0.35612500
 N                 -1.84233100   -0.09200500   -0.34956600
 C                  0.48411900    0.59567200    0.09332400
 H                  0.70097200    1.20639300    0.97490100
 N                  1.63189700   -0.25472300   -0.18405100
 H                  1.83523500   -0.87435200    0.59344800
 H                  0.32059100    1.27170300   -0.74654200
 H                  1.46555600   -0.83254100   -1.00134800
 H                 -0.80414300   -0.86453800    1.21816300
    Core  RigidRotor
     SymmetryFactor    1.000000000000000
    End
     Frequencies[1/cm]           21
108.91
313.96
358.17
427.84
769.98
822.12
912.75
928.74
1096.05
1125.66
1245.90
1342.55
1382.17
1479.10
1668.53
1717.76
2937.10
3044.11
3107.73
3498.34
3579.19
     ZeroEnergy[kJ/mol]        -180.90
     ElectronicLevels[1/cm]                     1
     0.0000000000000000        2.0000000000000000
 End
 End
 !***********************************
Barrier L1  REACS FC01
   RRHO
    Stoichiometry  C2N2H5
     Core PhaseSpaceTheory
            FragmentGeometry[angstrom]              7
 C                  0.05052800    0.70535200    0.00000000
```

```
 H                  0.59223700    1.06563400   -0.87850300
 H                 -0.94333700    1.18056700    0.00000000
 H                  0.59223700    1.06563400    0.87850300
 N                  0.05052800   -0.76080300    0.00000000
 H                 -0.44900100   -1.10916200    0.81210100
 H                 -0.44900100   -1.10916200   -0.81210100
      FragmentGeometry[angstrom]               2
 C                  0.00000000    0.00000000   -0.62766800
 N                  0.00000000    0.00000000    0.53800100
  SymmetryFactor     1.0000000000000000
          PotentialPrefactor[au]        94.4
          PotentialPowerExponent  6
      End
    Frequencies[1/cm]     16
316.27
851.39
979.67
1056.00
1170.62
1352.24
1461.12
1496.80
1518.88
1668.86
2152.08
2947.98
3051.48
3086.76
3488.87
3562.98
 ZeroEnergy[kJ/mol]            0.00
 ElectronicLevels[1/cm]                    1
  0.0000000000000000        2.0000000000000000
  End
Barrier TS0 FC01 RI
  RRHO
   Geometry[angstrom]            9
 C                  0.00000000    1.13383200    0.00000000
 N                  1.35605500    0.87199700    0.00000000
 C                 -1.13064900   -0.75223200    0.00000000
 N                 -0.45504300   -1.71237700    0.00000000
 H                 -0.39615700    1.55087000    0.92022400
 H                 -0.39615700    1.55087000   -0.92022400
 H                 -2.11415300   -0.30942200    0.00000000
 H                  1.69163900    0.40036800    0.83024300
 H                  1.69163900    0.40036800   -0.83024300
  Core  RigidRotor
    SymmetryFactor   1.0
  End
  Tunneling    Eckart
    ImaginaryFrequency[1/cm] 367.27
    WellDepth[kJ/mol]     36.0
    WellDepth[kJ/mol]     63.6
  End
  Frequencies[1/cm]            20
 62.30
```

```
160.79
286.91
397.00
620.62
753.71
754.32
776.79
943.39
993.56
1243.97
1352.35
1489.97
1672.67
1976.26
3106.77
3207.91
3240.18
3531.68
3621.98
  ZeroEnergy[kJ/mol]  -117.32
  ElectronicLevels[1/cm]                     1
       0        2
 End
 !************************************
Barrier TS1 RI P1
  RRHO
   Geometry[angstrom]            9
 C                -0.38083300   -1.19919900    0.00000000
 N                -1.50353400   -1.53720700    0.00000000
 C                 0.00000000    0.97709500    0.00000000
 H                -0.52965100    1.19378200    0.91914200
 N                 1.35088200    1.28243600    0.00000000
 H                 1.87045500    1.08368200    0.84246500
 H                -0.52965100    1.19378200   -0.91914200
 H                 1.87045500    1.08368200   -0.84246500
 H                 0.67195300   -1.43890100    0.00000000
  Core  RigidRotor
    SymmetryFactor   1.0
  End
  Tunneling    Eckart
    ImaginaryFrequency[1/cm]   339.79
    WellDepth[kJ/mol]          67.2
    WellDepth[kJ/mol]          27.2
  End
   Frequencies[1/cm]            20
47.13
175.78
335.20
352.64
559.08
609.78
754.45
790.32
934.94
972.71
1227.71
```

```
1328.82
1481.76
1662.58
1982.61
3134.67
3216.22
3239.94
3557.74
3660.87
  ZeroEnergy[kJ/mol]  -113.71
    ElectronicLevels[1/cm]                      1
       0        2
 End
 !************************************
Barrier TS2 RI P2
  RRHO
   Geometry[angstrom]            9
 C                -0.74374200    0.00743800    0.00000800
 N                -1.83837200   -0.39316500    0.00000500
 C                 0.60087800    0.61071200    0.00000300
 H                 0.67763900    1.26156500    0.87618400
 N                 1.72634900   -0.32216400   -0.00000600
 H                 1.68399000   -0.92523900    0.81537600
 H                -3.08189000    0.62574800   -0.00005100
 H                 0.67762900    1.26157000   -0.87617400
 H                 1.68397700   -0.92523800   -0.81538900
  Core  RigidRotor
    SymmetryFactor   1.0
  End
  Tunneling    Eckart
    ImaginaryFrequency[1/cm]   952.55
    WellDepth[kJ/mol]          123.9
    WellDepth[kJ/mol]          28.6
    End
   Frequencies[1/cm]            20
26.79
176.11
276.40
417.37
425.02
615.91
829.35
889.62
911.65
1096.45
1192.55
1358.22
1385.83
1457.88
1673.45
2210.83
3040.62
3076.78
3498.74
3576.03
  ZeroEnergy[kJ/mol]  -57.00
```

```
    ElectronicLevels[1/cm]                      1
       0        2
 End
End
```

CN attack to the NH_2 moiety
jChS

```
!***************************************************
!              GLOBAL SECTION
!***************************************************
!!!!!!!!!!!!!!!!!!!!!!!!!!!!!!!!!!!!!!!!!!!!!!!!!!!!
!
!
TemperatureList[K]                     30 40 50 60 70 80 90 100 110 120 130 140 150 160 170 180 190 200 220 240 260 280 300 350 400 450 500
PressureList[atm]                       1.e-8

!
!
EnergyStepOverTemperature               .2
ExcessEnergyOverTemperature             30
ModelEnergyLimit[kcal/mol]              400
!
CalculationMethod                        low-eigenvalue
!
WellCutoff                              20
ChemicalEigenvalueMax                   0.2
!
ReductionMethod                         diagonalization
!
AtomDistanceMin[bohr]                  1.3
!!
RateOutput                              ic01_ChS.out
!
!
!!!!!!!!!!!!!!!!!!!!!!!!!!!!!!!!!!!!!!!!!!!!!!!!!!!!
!***************************************************
!              MODEL SECTION
!***************************************************
!!!!!!!!!!!!!!!!!!!!!!!!!!!!!!!!!!!!!!!!!!!!!!!!!!!!
!
!
Model
!
  EnergyRelaxation
    Exponential
       Factor[1/cm]                     260
       Power                            0.875
       ExponentCutoff                   10
    End
!
   CollisionFrequency
```

```
   LennardJones
      Epsilons[K]                       127.697   1382.375
      Sigmas[angstrom]                    3.462   4.046
      Masses[amu]                       39.948 70.07
  End
!
!*************************************************
!
!***************************************************
!!!!!!!!!!!!!!!!!!!!!!!!!!!!!!!!!!!!!!!!!!!!!!!!!!!
!***************************************************
!  REACTANTS  - CH3NH2 + CN
!***************************************************
 Bimolecular REACS
 Fragment CH3NH2
 RRHO
 Geometry[angstrom]               7
 C                 -0.74064000    0.00000000    0.01360800
 H                 -1.12725000   -0.87638300   -0.50253800
 H                 -1.14607300    0.00000000    1.03050000
 H                 -1.12725000    0.87638400   -0.50253700
 N                  0.72026000    0.00000000   -0.07006200
 H                  1.10585300    0.81199300    0.39300800
 H                  1.10585300   -0.81199300    0.39300800
  Core  RigidRotor
  SymmetryFactor    1.0000000000000000
  End
     Frequencies[1/cm]         15
     300.20
     834.61
     978.18
     1064.10
     1178.30
     1354.60
     1472.48
     1517.20
     1536.05
     1672.27
     3006.41
     3092.76
     3129.63
     3519.95
     3601.33
  ZeroEnergy[kJ/mol]             0.
  ElectronicLevels[1/cm]                       1
   0.0000000000000000        1.0000000000000000
 !************************************
 End
!*********
 Fragment CN
 RRHO
 Geometry[angstrom]               2
 C                  0.00000000    0.00000000   -0.63181100
 N                  0.00000000    0.00000000    0.54143400
  Core  RigidRotor
  SymmetryFactor    1.0000000000000000
```

```
  End
     Frequencies[1/cm]            1
    2076.80
  ZeroEnergy[kJ/mol]            0.
  ElectronicLevels[1/cm]                      1
   0.0000000000000000        2.0000000000000000
  !************************************
 End
!*********
GroundEnergy[kJ/mol] 0.0
End
 Bimolecular P3
 Fragment CH3NH
 RRHO
 Geometry[angstrom]               6
  C                 -0.69085600    0.00887000    0.00000000
 H                 -1.05474600    0.55868200    0.87622200
 H                 -1.05474500    0.55864600   -0.87624400
 H                 -1.13400600   -0.98338300    0.00002000
 N                  0.74594800   -0.08039400    0.00000000
 H                  1.10494300    0.87746000    0.00000000
  Core  RigidRotor
  SymmetryFactor    1.0000000000000000
  End
     Frequencies[1/cm]            12
255.66
958.87
1010.57
1053.99
1343.98
1410.00
1498.21
1500.11
2985.70
3026.18
3127.98
3420.26
  ZeroEnergy[kJ/mol]            0.
  ElectronicLevels[1/cm]                      1
   0.0000000000000000        2.0000000000000000
    !************************************
 End
!*********
 Fragment HCN
 RRHO
 Geometry[angstrom]               3
  C                  0.00000000    0.00000000   -0.55868200
  H                  0.00000000    0.00000000   -1.62356200
  N                  0.00000000    0.00000000    0.59561500
  Core  RigidRotor
  SymmetryFactor    1.0000000000000000
  End
     Frequencies[1/cm]            4
    747.43
    747.43
    2124.86
```

```
    3444.80
  ZeroEnergy[kJ/mol]            0.
  ElectronicLevels[1/cm]                    1
   0.0000000000000000       1.0000000000000000
  !************************************
 End
!*********
GroundEnergy[kJ/mol] -111.90
End
!****************************************************
!  PRODUCT P4  - CH3NHCN + H
!****************************************************
 Bimolecular P4
 Fragment CH3NHCN
 RRHO
 Geometry[angstrom]              8
 C                 1.53244100   -0.37898500    0.02157500
 N                 0.43209200    0.58397800   -0.10011300
 C                -0.81909400    0.11839600   -0.00760600
 N                -1.90285100   -0.30468800    0.02081700
 H                 2.45946800    0.13032500   -0.22343000
 H                 1.60598600   -0.80817300    1.02109600
 H                 1.37601000   -1.17815600   -0.69645000
 H                 0.57376600    1.46450200    0.37003800
  Core  RigidRotor
  SymmetryFactor    1.0000000000000000
  End
     Frequencies[1/cm]           18
158.52
218.91
423.84
536.28
640.38
932.04
1152.59
1162.17
1204.99
1471.04
1475.34
1512.01
1535.38
2280.77
3055.05
3135.64
3169.35
3599.36
  ZeroEnergy[kJ/mol]            0.
  ElectronicLevels[1/cm]                    1
   0.0000000000000000       1.0000000000000000
    !************************************
 End
!*********
 Fragment HYDROGEN
  Atom
  Name H
  ElectronicLevels[1/cm]                    1
```

```
   0.0000000000000000        2.0000000000000000
    !************************************
 End
GroundEnergy[kJ/mol] -58.00
End
!***************************************************
!  PRODUCT P5  - NH2CN + CH3
!***************************************************
Bimolecular P5
 Fragment NH2CN
 RRHO
 Geometry[angstrom]               5
 N                 -0.08716600   -1.12179000    0.00000000
 C                  0.00000000    0.21953600    0.00000000
 N                  0.01641700    1.38102100    0.00000000
 H                  0.24762200   -1.56591400    0.84100100
 H                  0.24762200   -1.56591400   -0.84100100
     Core  RigidRotor
     SymmetryFactor    1.0000000000000000
     End
 Frequencies[1/cm]            9
 404.14
 486.58
 594.16
 1083.11
 1204.01
 1643.46
 2300.28
 3564.12
 3659.42
  ZeroEnergy[kJ/mol]            0.
  ElectronicLevels[1/cm]                      1
   0.0000000000000000        1.0000000000000000
    !************************************
 End
!*********
 Fragment CH3
 RRHO
 Geometry[angstrom]               4
   H                  0.00000000    1.07590000    0.00000000
 C                  0.00000000    0.00000000    0.00000000
 H                 -0.93175700   -0.53795000    0.00000000
 H                  0.93175700   -0.53795000    0.00000000
  Core  RigidRotor
  SymmetryFactor    6.0
  End
     Frequencies[1/cm]          6
    526.96
    1426.53
    1426.53
    3138.66
    3319.82
    3319.82
  ZeroEnergy[kJ/mol]            0.
  ElectronicLevels[1/cm]                      1
   0.0000000000000000        2.0000000000000000
```

```
  !************************************
 End
!*********
GroundEnergy[kJ/mol] -142.70
End
 !************************************
Well IC
 Species
 RRHO     ! transition state
 Geometry[angstrom]              9
 C                 0.29460700   -1.50040600    0.00000000
 N                -0.86441000   -0.61200300    0.00000000
 C                -0.18734900    1.31574300    0.00000000
 N                 0.92703100    1.68660400    0.00000000
 H                 0.00000000   -2.54992400    0.00000000
 H                 0.89462600   -1.28692000   -0.87827200
 H                 0.89462600   -1.28692000    0.87827200
 H                -1.43557700   -0.64523300   -0.83215800
 H                -1.43557700   -0.64523300    0.83215800
  Core  RigidRotor
  SymmetryFactor    1.000000000000000
  End
     Frequencies[1/cm]            21
     24.78
     132.03
     172.11
     264.31
     436.02
     536.71
     839.50
     962.22
     1049.53
     1155.95
     1321.96
     1469.63
     1510.06
     1514.86
     1651.20
     2066.50
     3060.30
     3148.70
     3181.49
     3541.98
     3642.59
     ZeroEnergy[kJ/mol]         -65.60
     ElectronicLevels[1/cm]                      1
      0.0000000000000000        2.0000000000000000
       End
        End
 !************************************
Well FC02
 Species
 RRHO     ! transition state
 Geometry[angstrom]              9
 C                 1.66001200    0.06662700   -0.00000400
 N                 2.79086200   -0.17012900    0.00000300
```

```
 C                 -2.42608800   -0.48814000    0.00000100
 N                 -1.45407200    0.57096900   -0.00000200
 H                 -3.08074200   -0.41850500    0.87608300
 H                 -3.08076300   -0.41848500   -0.87606400
 H                 -1.93627100   -1.45772100   -0.00001700
 H                  0.60167200    0.28371700   -0.00000900
 H                 -1.95618900    1.46047500    0.00002200
  Core  RigidRotor
  SymmetryFactor    1.000000000000000
  End
     Frequencies[1/cm]           21
     37.26
     71.02
     153.41
     155.12
     188.95
     342.93
     918.96
     927.71
     967.71
     1016.71
     1068.77
     1344.50
     1409.88
     1494.32
     1504.55
     2104.21
     2994.20
     3037.06
     3136.97
     3219.83
     3442.82
     ZeroEnergy[kJ/mol]        -134.10
  ElectronicLevels[1/cm]                      1
   0.0000000000000000        2.0000000000000000
 End
 End
Barrier L2  REACS IC
   RRHO
    Stoichiometry  C2N2H5
    Core PhaseSpaceTheory
      FragmentGeometry[angstrom]              7
 C                 -0.74064000    0.00000000    0.01360800
 H                 -1.12725000   -0.87638300   -0.50253800
 H                 -1.14607300    0.00000000    1.03050000
 H                 -1.12725000    0.87638400   -0.50253700
 N                  0.72026000    0.00000000   -0.07006200
 H                  1.10585300    0.81199300    0.39300800
 H                  1.10585300   -0.81199300    0.39300800
      FragmentGeometry[angstrom]              2
 C                  0.00000000    0.00000000   -0.63181100
 N                  0.00000000    0.00000000    0.54143400
  SymmetryFactor     1.0000000000000000
          PotentialPrefactor[au]       94.4
          PotentialPowerExponent  6
      End
```

```
    Frequencies[1/cm]     16
     300.20
     834.61
     978.18
     1064.10
     1178.30
     1354.60
     1472.48
     1517.20
     1536.05
     1672.27
     2076.80
     3006.41
     3092.76
     3129.63
     3519.95
     3601.33
 ZeroEnergy[kJ/mol]            0.00
 ElectronicLevels[1/cm]                   1
  0.0000000000000000        2.0000000000000000
  End
Barrier L4  FC02 P3
   RRHO
    Stoichiometry  C2N2H5
    Core PhaseSpaceTheory
      FragmentGeometry[angstrom]               6
        C                 -0.69085600    0.00887000    0.00000000
  H                 -1.05474600    0.55868200    0.87622200
  H                 -1.05474500    0.55864600   -0.87624400
  H                 -1.13400600   -0.98338300    0.00002000
  N                  0.74594800   -0.08039400    0.00000000
  H                  1.10494300    0.87746000    0.00000000
      FragmentGeometry[angstrom]               3
         C                  0.00000000    0.00000000   -0.55868200
    H                  0.00000000    0.00000000   -1.62356200
    N                  0.00000000    0.00000000    0.59561500
  SymmetryFactor     1.0000000000000000
          PotentialPrefactor[au]        3703.38
          PotentialPowerExponent  6
      End
    Frequencies[1/cm]     16
    255.66
    747.43
    747.43
    958.87
    1010.57
    1053.99
    1343.98
    1410.00
    1498.21
    1500.11
    2124.86
    2985.70
    3026.18
    3127.98
    3420.26
```

```
   3444.80
 ZeroEnergy[kJ/mol]            -111.90
 ElectronicLevels[1/cm]                   1
  0.0000000000000000        2.0000000000000000
  End
 !************************************
Barrier TS3 IC FC02
  RRHO
   Geometry[angstrom]            9
    C                 -1.33741800    0.19176100    0.17890200
 N                 -2.34945200   -0.30513400   -0.12626400
 C                  1.70239700   -0.56940300    0.00091500
 N                  1.07127000    0.73480900    0.02464900
 H                  1.23907200   -1.25220200   -0.71168700
 H                  2.74469900   -0.43572600   -0.28906700
 H                  1.66084400   -1.00657900    0.99315100
 H                  0.12879500    0.79015700    0.53223000
 H                  0.98398500    1.16247500   -0.89221900
  Core  RigidRotor
    SymmetryFactor   1.0
  End
  Tunneling    Eckart
    ImaginaryFrequency[1/cm]   535.14
    WellDepth[kJ/mol]          30.2
    WellDepth[kJ/mol]          98.7
    End
   Frequencies[1/cm]            20
       77.52
       103.17
       147.98
       227.06
       350.41
       713.50
       973.18
       1052.64
       1105.93
       1315.92
       1452.23
       1489.76
       1514.96
       1583.50
       2126.15
       2478.08
       3049.19
       3107.07
       3166.10
       3535.45
  ZeroEnergy[kJ/mol]  -35.40
    ElectronicLevels[1/cm]                      1
       0        2
 End
 !************************************
Barrier TS4 IC P4
  RRHO
   Geometry[angstrom]            9
  C                  1.52806200   -0.43812800    0.02657200
```

```
 N                 0.41957900    0.56413300   -0.02968000
 C                -0.84204700   -0.01160500   -0.20031800
 N                -1.94366900   -0.25912100    0.11660800
 H                 2.44466700    0.10873900    0.22491700
 H                 1.31521500   -1.11200800    0.84889100
 H                 1.58828300   -0.98581600   -0.90655900
 H                 0.61920500    1.31471200    1.04264900
 H                 0.58516800    1.23768500   -0.77592400
  Core  RigidRotor
    SymmetryFactor   1.0
  End
  Tunneling    Eckart
    ImaginaryFrequency[1/cm]   1434.92
    WellDepth[kJ/mol]         89.5
    WellDepth[kJ/mol]         81.9
    End
   Frequencies[1/cm]            20
   174.82
   199.88
   382.91
   537.21
   705.43
   789.04
   893.61
   1082.33
   1092.11
   1172.72
   1214.86
   1412.44
   1452.21
   1503.22
   1517.79
   2155.85
   3091.68
   3184.78
   3196.63
   3455.44
  ZeroEnergy[kJ/mol]  23.90
    ElectronicLevels[1/cm]                      1
       0        2
 End
  !*************************************
Barrier TS5 IC P5
  RRHO
   Geometry[angstrom]            9
   C                -1.74878000    0.21620800    0.00000000
 N                 0.00000000    0.78377900    0.00000000
 C                 1.11650200   -0.14313300    0.00000000
 N                 1.36926600   -1.29687500    0.00000000
 H                -2.34597400    1.11780000    0.00000000
 H                -1.80704600   -0.36970000   -0.90503900
 H                -1.80704600   -0.36970000    0.90503900
 H                 0.08443400    1.38741300   -0.81741300
 H                 0.08443400    1.38741300    0.81741300
  Core  RigidRotor
    SymmetryFactor   1.0
```

```
  End
  Tunneling    Eckart
    ImaginaryFrequency[1/cm]   791.49
    WellDepth[kJ/mol]         87.0
    WellDepth[kJ/mol]         164.1
    End
   Frequencies[1/cm]            20
   86.08
   151.75
   242.52
   502.17
   577.01
   656.91
   863.11
   874.81
   1114.14
   1144.58
   1214.79
   1448.80
   1460.47
   1601.47
   2054.94
   3113.24
   3252.34
   3260.97
   3419.62
   3507.47
  ZeroEnergy[kJ/mol]  21.40
    ElectronicLevels[1/cm]                      1
       0        2
 End
End
```

CBS-CVH

```
!***************************************************
!               GLOBAL SECTION
!***************************************************
!!!!!!!!!!!!!!!!!!!!!!!!!!!!!!!!!!!!!!!!!!!!!!!!!!!!
!
!
TemperatureList[K]                     30 40 50 60 70 80 90 100 110 120 130 140 150 160 170 180 190 200 220 240 260 280 300 350 400 450 500
PressureList[atm]                       1.e-8
!
!
EnergyStepOverTemperature               .2
ExcessEnergyOverTemperature             30
ModelEnergyLimit[kcal/mol]              400
!
CalculationMethod                        low-eigenvalue
!
WellCutoff                              20
ChemicalEigenvalueMax                   0.2
!
ReductionMethod                         diagonalization
```

```
AtomDistanceMin[bohr]                  1.3
!!
RateOutput                              ic01_HEATlike.out
!
!
!!!!!!!!!!!!!!!!!!!!!!!!!!!!!!!!!!!!!!!!!!!!!!!!!!!
!**************************************************
!               MODEL SECTION
!**************************************************
!!!!!!!!!!!!!!!!!!!!!!!!!!!!!!!!!!!!!!!!!!!!!!!!!!!
!
!
Model
!
  EnergyRelaxation
    Exponential
       Factor[1/cm]                     260
       Power                            0.875
       ExponentCutoff                   10
    End
!
   CollisionFrequency
    LennardJones
       Epsilons[K]                       127.697   1382.375
       Sigmas[angstrom]                    3.462   4.046
       Masses[amu]                       39.948 70.07
   End
!
!*************************************************
!
!**************************************************
!!!!!!!!!!!!!!!!!!!!!!!!!!!!!!!!!!!!!!!!!!!!!!!!!!!
!**************************************************
!  REACTANTS  - CH3NH2 + CN
!**************************************************
 Bimolecular REACS
 Fragment CH3NH2
 RRHO
 Geometry[angstrom]               7
 C                 -0.74064000    0.00000000    0.01360800
 H                 -1.12725000   -0.87638300   -0.50253800
 H                 -1.14607300    0.00000000    1.03050000
 H                 -1.12725000    0.87638400   -0.50253700
 N                  0.72026000    0.00000000   -0.07006200
 H                  1.10585300    0.81199300    0.39300800
 H                  1.10585300   -0.81199300    0.39300800
  Core  RigidRotor
  SymmetryFactor    1.0000000000000000
  End
     Frequencies[1/cm]         15
     300.20
     834.61
     978.18
     1064.10
     1178.30
     1354.60
```

```
    1472.48
    1517.20
    1536.05
    1672.27
    3006.41
    3092.76
    3129.63
    3519.95
    3601.33
  ZeroEnergy[kJ/mol]            0.
  ElectronicLevels[1/cm]                      1
   0.0000000000000000        1.0000000000000000
 !************************************
 End
!*********
 Fragment CN
 RRHO
 Geometry[angstrom]              2
 C                 0.00000000    0.00000000   -0.63181100
 N                 0.00000000    0.00000000    0.54143400
  Core  RigidRotor
  SymmetryFactor    1.0000000000000000
  End
    Frequencies[1/cm]           1
   2076.80
  ZeroEnergy[kJ/mol]            0.
  ElectronicLevels[1/cm]                      1
   0.0000000000000000        2.0000000000000000
  !************************************
 End
!*********
GroundEnergy[kJ/mol] 0.0
End
!****************************************************
!  PRODUCT P3  - CH3NH + HCN
!****************************************************
 Bimolecular P3
 Fragment CH3NH
 RRHO
 Geometry[angstrom]              6
  C                -0.69085600    0.00887000    0.00000000
 H                -1.05474600    0.55868200    0.87622200
 H                -1.05474500    0.55864600   -0.87624400
 H                -1.13400600   -0.98338300    0.00002000
 N                 0.74594800   -0.08039400    0.00000000
 H                 1.10494300    0.87746000    0.00000000
  Core  RigidRotor
  SymmetryFactor    1.0000000000000000
  End
    Frequencies[1/cm]           12
255.66
958.87
1010.57
1053.99
1343.98
1410.00
```

```
1498.21
1500.11
2985.70
3026.18
3127.98
3420.26
  ZeroEnergy[kJ/mol]            0.
  ElectronicLevels[1/cm]                     1
   0.0000000000000000       2.0000000000000000
    !************************************
 End
!*********
 Fragment HCN
 RRHO
 Geometry[angstrom]              3
  C                 0.00000000    0.00000000   -0.55868200
  H                 0.00000000    0.00000000   -1.62356200
  N                 0.00000000    0.00000000    0.59561500
  Core  RigidRotor
  SymmetryFactor    1.0000000000000000
  End
     Frequencies[1/cm]           4
    747.43
    747.43
    2124.86
    3444.80
  ZeroEnergy[kJ/mol]            0.
  ElectronicLevels[1/cm]                     1
   0.0000000000000000       1.0000000000000000
  !************************************
 End
!*********
GroundEnergy[kJ/mol] -110.70
End
!****************************************************
!  PRODUCT P4  - CH3NHCN + H
!****************************************************
 Bimolecular P4
 Fragment CH3NHCN
 RRHO
 Geometry[angstrom]              8
 C                 1.53244100   -0.37898500    0.02157500
 N                 0.43209200    0.58397800   -0.10011300
 C                -0.81909400    0.11839600   -0.00760600
 N                -1.90285100   -0.30468800    0.02081700
 H                 2.45946800    0.13032500   -0.22343000
 H                 1.60598600   -0.80817300    1.02109600
 H                 1.37601000   -1.17815600   -0.69645000
 H                 0.57376600    1.46450200    0.37003800
  Core  RigidRotor
  SymmetryFactor    1.0000000000000000
  End
     Frequencies[1/cm]           18
158.52
218.91
423.84
```

```
536.28
640.38
932.04
1152.59
1162.17
1204.99
1471.04
1475.34
1512.01
1535.38
2280.77
3055.05
3135.64
3169.35
3599.36
  ZeroEnergy[kJ/mol]             0.
  ElectronicLevels[1/cm]                      1
   0.0000000000000000        1.0000000000000000
    !************************************
 End
!*********
 Fragment HYDROGEN
  Atom
  Name H
  ElectronicLevels[1/cm]                      1
   0.0000000000000000        2.0000000000000000
    !************************************
 End
GroundEnergy[kJ/mol] -56.70
End
!****************************************************
!  PRODUCT P5  - NH2CN + CH3
!****************************************************
Bimolecular P5
 Fragment NH2CN
 RRHO
 Geometry[angstrom]               5
 N                 -0.08716600   -1.12179000    0.00000000
 C                  0.00000000    0.21953600    0.00000000
 N                  0.01641700    1.38102100    0.00000000
 H                  0.24762200   -1.56591400    0.84100100
 H                  0.24762200   -1.56591400   -0.84100100
     Core  RigidRotor
     SymmetryFactor    1.0000000000000000
     End
 Frequencies[1/cm]            9
 404.14
 486.58
 594.16
 1083.11
 1204.01
 1643.46
 2300.28
 3564.12
 3659.42
  ZeroEnergy[kJ/mol]             0.
```

```
 ElectronicLevels[1/cm]                    1
  0.0000000000000000       1.0000000000000000
   !************************************
 End
!*********
 Fragment CH3
 RRHO
 Geometry[angstrom]              4
   H                  0.00000000    1.07590000    0.00000000
 C                  0.00000000    0.00000000    0.00000000
 H                 -0.93175700   -0.53795000    0.00000000
 H                  0.93175700   -0.53795000    0.00000000
  Core  RigidRotor
  SymmetryFactor    6.0
  End
     Frequencies[1/cm]         6
    526.96
    1426.53
    1426.53
    3138.66
    3319.82
    3319.82
  ZeroEnergy[kJ/mol]            0.
  ElectronicLevels[1/cm]                    1
   0.0000000000000000       2.0000000000000000
  !************************************
 End
!*********
GroundEnergy[kJ/mol] -140.40
End
 !************************************
Well IC
 Species
 RRHO     ! transition state
 Geometry[angstrom]              9
 C                  0.29460700   -1.50040600    0.00000000
 N                 -0.86441000   -0.61200300    0.00000000
 C                 -0.18734900    1.31574300    0.00000000
 N                  0.92703100    1.68660400    0.00000000
 H                  0.00000000   -2.54992400    0.00000000
 H                  0.89462600   -1.28692000   -0.87827200
 H                  0.89462600   -1.28692000    0.87827200
 H                 -1.43557700   -0.64523300   -0.83215800
 H                 -1.43557700   -0.64523300    0.83215800
  Core  RigidRotor
  SymmetryFactor    1.000000000000000
  End
     Frequencies[1/cm]           21
     24.78
     132.03
     172.11
     264.31
     436.02
     536.71
     839.50
     962.22
```

```
    1049.53
    1155.95
    1321.96
    1469.63
    1510.06
    1514.86
    1651.20
    2066.50
    3060.30
    3148.70
    3181.49
    3541.98
    3642.59
    ZeroEnergy[kJ/mol]         -63.60
    ElectronicLevels[1/cm]                      1
     0.0000000000000000        2.0000000000000000
      End
       End
 !************************************
Well FC02
 Species
 RRHO     ! transition state
 Geometry[angstrom]              9
 C                 1.66001200    0.06662700   -0.00000400
 N                 2.79086200   -0.17012900    0.00000300
 C                -2.42608800   -0.48814000    0.00000100
 N                -1.45407200    0.57096900   -0.00000200
 H                -3.08074200   -0.41850500    0.87608300
 H                -3.08076300   -0.41848500   -0.87606400
 H                -1.93627100   -1.45772100   -0.00001700
 H                 0.60167200    0.28371700   -0.00000900
 H                -1.95618900    1.46047500    0.00002200
  Core  RigidRotor
  SymmetryFactor    1.000000000000000
  End
    Frequencies[1/cm]            21
    37.26
    71.02
    153.41
    155.12
    188.95
    342.93
    918.96
    927.71
    967.71
    1016.71
    1068.77
    1344.50
    1409.88
    1494.32
    1504.55
    2104.21
    2994.20
    3037.06
    3136.97
    3219.83
```

```
     3442.82
     ZeroEnergy[kJ/mol]        -133.00
  ElectronicLevels[1/cm]                      1
   0.0000000000000000        2.0000000000000000
 End
 End
!************************************
Barrier L2  REACS IC
   RRHO
    Stoichiometry  C2N2H5
    Core PhaseSpaceTheory
      FragmentGeometry[angstrom]              7
 C                -0.74064000    0.00000000    0.01360800
 H                -1.12725000   -0.87638300   -0.50253800
 H                -1.14607300    0.00000000    1.03050000
 H                -1.12725000    0.87638400   -0.50253700
 N                 0.72026000    0.00000000   -0.07006200
 H                 1.10585300    0.81199300    0.39300800
 H                 1.10585300   -0.81199300    0.39300800
      FragmentGeometry[angstrom]              2
 C                 0.00000000    0.00000000   -0.63181100
 N                 0.00000000    0.00000000    0.54143400
  SymmetryFactor     1.0000000000000000
          PotentialPrefactor[au]        94.4
          PotentialPowerExponent  6
      End
    Frequencies[1/cm]     16
     300.20
     834.61
     978.18
     1064.10
     1178.30
     1354.60
     1472.48
     1517.20
     1536.05
     1672.27
     2076.80
     3006.41
     3092.76
     3129.63
     3519.95
     3601.33
 ZeroEnergy[kJ/mol]            0.00
 ElectronicLevels[1/cm]                   1
  0.0000000000000000        2.0000000000000000
  End
Barrier L4  FC02 P3
   RRHO
    Stoichiometry  C2N2H5
    Core PhaseSpaceTheory
      FragmentGeometry[angstrom]              6
        C                 -0.69085600    0.00887000    0.00000000
  H                 -1.05474600    0.55868200    0.87622200
  H                 -1.05474500    0.55864600   -0.87624400
  H                 -1.13400600   -0.98338300    0.00002000
```

```
  N                  0.74594800   -0.08039400    0.00000000
  H                  1.10494300    0.87746000    0.00000000
      FragmentGeometry[angstrom]              3
         C                  0.00000000    0.00000000   -0.55868200
    H                  0.00000000    0.00000000   -1.62356200
    N                  0.00000000    0.00000000    0.59561500
  SymmetryFactor     1.0000000000000000
        PotentialPrefactor[au]        3703.38
        PotentialPowerExponent  6
      End
    Frequencies[1/cm]     16
    255.66
    747.43
    747.43
    958.87
    1010.57
    1053.99
    1343.98
    1410.00
    1498.21
    1500.11
    2124.86
    2985.70
    3026.18
    3127.98
    3420.26
    3444.80
 ZeroEnergy[kJ/mol]            -110.70
 ElectronicLevels[1/cm]                  1
  0.0000000000000000        2.0000000000000000
  End
 !*************************************
Barrier TS3 IC FC02
  RRHO
   Geometry[angstrom]            9
    C                 -1.33741800    0.19176100    0.17890200
 N                -2.34945200   -0.30513400   -0.12626400
 C                 1.70239700   -0.56940300    0.00091500
 N                 1.07127000    0.73480900    0.02464900
 H                 1.23907200   -1.25220200   -0.71168700
 H                 2.74469900   -0.43572600   -0.28906700
 H                 1.66084400   -1.00657900    0.99315100
 H                 0.12879500    0.79015700    0.53223000
 H                 0.98398500    1.16247500   -0.89221900
  Core  RigidRotor
    SymmetryFactor   1.0
  End
  Tunneling    Eckart
    ImaginaryFrequency[1/cm]   535.14
    WellDepth[kJ/mol]          27.2
    WellDepth[kJ/mol]          96.6
    End
   Frequencies[1/cm]            20
       77.52
       103.17
       147.98
```

```
      227.06
      350.41
      713.50
      973.18
      1052.64
      1105.93
      1315.92
      1452.23
      1489.76
      1514.96
      1583.50
      2126.15
      2478.08
      3049.19
      3107.07
      3166.10
      3535.45
  ZeroEnergy[kJ/mol]  -36.40
    ElectronicLevels[1/cm]                      1
      0        2
 End
 !*************************************
Barrier TS4 IC P4
  RRHO
   Geometry[angstrom]            9
  C                  1.52806200   -0.43812800    0.02657200
 N                  0.41957900    0.56413300   -0.02968000
 C                 -0.84204700   -0.01160500   -0.20031800
 N                 -1.94366900   -0.25912100    0.11660800
 H                  2.44466700    0.10873900    0.22491700
 H                  1.31521500   -1.11200800    0.84889100
 H                  1.58828300   -0.98581600   -0.90655900
 H                  0.61920500    1.31471200    1.04264900
 H                  0.58516800    1.23768500   -0.77592400
  Core  RigidRotor
    SymmetryFactor   1.0
  End
  Tunneling    Eckart
    ImaginaryFrequency[1/cm]   1434.92
    WellDepth[kJ/mol]         87.6
    WellDepth[kJ/mol]         80.7
    End
   Frequencies[1/cm]            20
   174.82
   199.88
   382.91
   537.21
   705.43
   789.04
   893.61
   1082.33
   1092.11
   1172.72
   1214.86
   1412.44
   1452.21
```

```
  1503.22
  1517.79
  2155.85
  3091.68
  3184.78
  3196.63
  3455.44
 ZeroEnergy[kJ/mol]  24.00
  ElectronicLevels[1/cm]                  1
     0       2
End
 !************************************
Barrier TS5 IC P5
 RRHO
  Geometry[angstrom]           9
  C                 -1.74878000    0.21620800    0.00000000
N                   0.00000000    0.78377900    0.00000000
C                   1.11650200   -0.14313300    0.00000000
N                   1.36926600   -1.29687500    0.00000000
H                  -2.34597400    1.11780000    0.00000000
H                  -1.80704600   -0.36970000   -0.90503900
H                  -1.80704600   -0.36970000    0.90503900
H                   0.08443400    1.38741300   -0.81741300
H                   0.08443400    1.38741300    0.81741300
 Core  RigidRotor
   SymmetryFactor   1.0
 End
 Tunneling    Eckart
   ImaginaryFrequency[1/cm]   791.49
   WellDepth[kJ/mol]         86.5
   WellDepth[kJ/mol]         163.3
   End
  Frequencies[1/cm]           20
  86.08
  151.75
  242.52
  502.17
  577.01
  656.91
  863.11
  874.81
  1114.14
  1144.58
  1214.79
  1448.80
  1460.47
  1601.47
  2054.94
  3113.24
  3252.34
  3260.97
  3419.62
  3507.47
 ZeroEnergy[kJ/mol]  22.90
  ElectronicLevels[1/cm]                  1
     0       2
```

```
 End
End
```

CBS-QB3

```
!**************************************************
!              GLOBAL SECTION
!**************************************************
!!!!!!!!!!!!!!!!!!!!!!!!!!!!!!!!!!!!!!!!!!!!!!!!!!!
!
!
TemperatureList[K]                     30 40 50 60 70 80 90 100 110 120 130 140 150 160 170 180 190 200 220 240 260 280 300 350 400 450 500
PressureList[atm]                       1.e-8
!
!
EnergyStepOverTemperature               .2
ExcessEnergyOverTemperature             30
ModelEnergyLimit[kcal/mol]              400
!
CalculationMethod                        low-eigenvalue
!
WellCutoff                              20
ChemicalEigenvalueMax                   0.2
!
ReductionMethod                         diagonalization
!
AtomDistanceMin[bohr]                  1.3
!!
RateOutput                              ic01_CBSQB3.out
!
!
!!!!!!!!!!!!!!!!!!!!!!!!!!!!!!!!!!!!!!!!!!!!!!!!!!!
!**************************************************
!              MODEL SECTION
!**************************************************
!!!!!!!!!!!!!!!!!!!!!!!!!!!!!!!!!!!!!!!!!!!!!!!!!!!
!
!
Model
!
  EnergyRelaxation
    Exponential
       Factor[1/cm]                     260
       Power                            0.875
       ExponentCutoff                   10
    End
!
   CollisionFrequency
    LennardJones
       Epsilons[K]                        127.697   1382.375
       Sigmas[angstrom]                     3.462   4.046
       Masses[amu]                        39.948 70.07
   End
!
!*************************************************
```

```
!
!**************************************************
!!!!!!!!!!!!!!!!!!!!!!!!!!!!!!!!!!!!!!!!!!!!!!!!!!!
!**************************************************
!  REACTANTS  - CH3NH2 + CN
!**************************************************
 Bimolecular REACS
 Fragment CH3NH2
 RRHO
 Geometry[angstrom]              7
  C                  0.05052800    0.70535200    0.00000000
 H                  0.59223700    1.06563400   -0.87850300
 H                 -0.94333700    1.18056700    0.00000000
 H                  0.59223700    1.06563400    0.87850300
 N                  0.05052800   -0.76080300    0.00000000
 H                 -0.44900100   -1.10916200    0.81210100
 H                 -0.44900100   -1.10916200   -0.81210100
  Core  RigidRotor
  SymmetryFactor    1.0000000000000000
  End
     Frequencies[1/cm]         15
316.27
851.39
979.67
1056.00
1170.62
1352.24
1461.12
1496.80
1518.88
1668.86
2947.98
3051.48
3086.76
3488.87
3562.98
  ZeroEnergy[kJ/mol]             0.
  ElectronicLevels[1/cm]                     1
   0.0000000000000000        1.0000000000000000
 !************************************
 End
!*********
 Fragment CN
 RRHO
 Geometry[angstrom]              2
 C                  0.00000000    0.00000000   -0.62766800
 N                  0.00000000    0.00000000    0.53800100
  Core  RigidRotor
  SymmetryFactor    1.0000000000000000
  End
     Frequencies[1/cm]            1
    2152.08
  ZeroEnergy[kJ/mol]             0.
  ElectronicLevels[1/cm]                     1
   0.0000000000000000        2.0000000000000000
  !************************************
```

```
 End
!*********
GroundEnergy[kJ/mol] 0.0
End
!**************************************************
!  PRODUCT P3  - CH3NH + HCN
!**************************************************
 Bimolecular P3
 Fragment CH3NH
 RRHO
 Geometry[angstrom]               6
 C                 -0.62788900   -0.01188200    0.00000000
 H                 -0.97212900   -0.58181800   -0.87879400
 H                 -0.97212600   -0.58178500    0.87881800
 H                 -1.12710100    0.95935900   -0.00001700
 N                  0.80507200    0.15292000   -0.00000200
 H                  1.20318700   -0.79490500    0.00000700
  Core  RigidRotor
  SymmetryFactor    1.0000000000000000
  End
     Frequencies[1/cm]            12
256.20
949.26
1008.25
1042.84
1341.71
1394.87
1479.27
1486.82
2939.89
2972.35
3082.63
3370.22
  ZeroEnergy[kJ/mol]            0.
  ElectronicLevels[1/cm]                      1
   0.0000000000000000        2.0000000000000000
    !************************************
 End
!*********
 Fragment HCN
 RRHO
 Geometry[angstrom]               3
 C                  0.00000000    0.00000000   -0.49814100
 H                  0.00000000    0.00000000   -1.56440500
 N                  0.00000000    0.00000000    0.65046400
  Core  RigidRotor
  SymmetryFactor    1.0000000000000000
  End
     Frequencies[1/cm]            4
785.56
785.56
2200.47
3457.47
  ZeroEnergy[kJ/mol]            0.
  ElectronicLevels[1/cm]                      1
   0.0000000000000000        1.0000000000000000
```

```
  !************************************
 End
!*********
GroundEnergy[kJ/mol] -113.03
End
!***************************************************
!  PRODUCT P4  - CH3NHCN + H
!***************************************************
 Bimolecular P4
 Fragment CH3NHCN
 RRHO
 Geometry[angstrom]               8
 C                  1.54537800   -0.37046500    0.02081200
 N                  0.42494100    0.57313700   -0.09318200
 C                 -0.82589600    0.11111900   -0.00754900
 N                 -1.90883200   -0.30115300    0.02014500
 H                  2.46023400    0.14566000   -0.27121100
 H                  1.66201800   -0.77180700    1.03325800
 H                  1.38190500   -1.19686900   -0.67061600
 H                  0.56618800    1.47520500    0.34025300
  Core  RigidRotor
  SymmetryFactor    1.0000000000000000
  End
     Frequencies[1/cm]            18
150.70
223.65
412.21
542.28
636.06
929.71
1140.20
1161.77
1203.81
1463.06
1469.04
1494.25
1521.31
2342.83
3018.44
3099.67
3134.52
3585.82
  ZeroEnergy[kJ/mol]            0.
  ElectronicLevels[1/cm]                      1
   0.0000000000000000        1.0000000000000000
    !************************************
 End
!*********
 Fragment HYDROGEN
  Atom
  Name H
  ElectronicLevels[1/cm]                      1
   0.0000000000000000        2.0000000000000000
    !************************************
 End
GroundEnergy[kJ/mol] -63.09
```

```
End
!***************************************************
!  PRODUCT P5  - NH2CN + CH3
!***************************************************
Bimolecular P5
 Fragment NH2CN
 RRHO
 Geometry[angstrom]               5
 N                 -0.09084700   -1.11782700    0.00000000
 C                  0.00000000    0.22085100    0.00000000
 N                  0.02567200    1.37768600    0.00000000
 H                  0.22811300   -1.57206300    0.84494500
 H                  0.22811300   -1.57206300   -0.84494500
     Core  RigidRotor
     SymmetryFactor    1.0000000000000000
     End
 Frequencies[1/cm]            9
 415.74
491.92
572.53
1096.32
1197.75
1636.02
2361.64
3550.73
3641.60
  ZeroEnergy[kJ/mol]             0.
  ElectronicLevels[1/cm]                      1
   0.0000000000000000        1.0000000000000000
    !************************************
 End
!*********
 Fragment CH3
 RRHO
 Geometry[angstrom]               4
 H                  0.00000000    1.08045600    0.00000000
 C                  0.00000000    0.00000000    0.00000000
 H                  0.93570200   -0.54022800    0.00000000
 H                 -0.93570200   -0.54022800    0.00000000
  Core  RigidRotor
  SymmetryFactor    6.0
  End
     Frequencies[1/cm]          6
505.11
1403.09
1403.09
3103.65
3282.67
3282.67
  ZeroEnergy[kJ/mol]             0.
  ElectronicLevels[1/cm]                      1
   0.0000000000000000        2.0000000000000000
  !************************************
 End
!*********
GroundEnergy[kJ/mol] -138.33
```

```
End
 !************************************
Well IC
 Species
 RRHO     ! transition state
 Geometry[angstrom]               9
 C                 1.40985800    0.68350600    0.00000000
 N                 0.00000000    1.07029000    0.00000000
 C                -1.18538600   -0.65684100    0.00000000
 N                -0.85538900   -1.77768800    0.00000000
 H                 2.07361500    1.55497200    0.00000000
 H                 1.60558700    0.07397300    0.88102200
 H                 1.60558700    0.07397300   -0.88102200
 H                -0.32195000    1.54443700    0.83450600
 H                -0.32195000    1.54443700   -0.83450600
  Core  RigidRotor
  SymmetryFactor    1.000000000000000
  End
     Frequencies[1/cm]            21
12.91
106.76
164.75
232.66
406.04
526.42
814.73
960.00
1044.02
1141.45
1313.03
1456.10
1493.90
1497.40
1646.27
2118.95
3020.96
3114.27
3148.60
3535.84
3632.78
     ZeroEnergy[kJ/mol]         -69.14
     ElectronicLevels[1/cm]                       1
      0.0000000000000000        2.0000000000000000
       End
        End
 !************************************
Well FC02
 Species
 RRHO     ! transition state
 Geometry[angstrom]               9
 C                 1.83345300    0.03434200   -0.00000200
 N                 2.96465600   -0.17257200   -0.00001300
 C                -2.33316100   -0.42244100    0.00001300
 N                -1.27398200    0.55184600   -0.00000200
 H                -2.98601300   -0.30103800    0.87882600
 H                -2.98600700   -0.30107300   -0.87881000
```

```
 H                 -1.92733300   -1.43508900    0.00003500
 H                  0.76705300    0.22778900    0.00000800
 H                 -1.70417000    1.48309400   -0.00002200
  Core  RigidRotor
  SymmetryFactor    1.000000000000000
  End
     Frequencies[1/cm]           21
48.73
71.19
156.70
157.47
202.19
351.47
940.25
959.26
966.32
1014.49
1064.32
1337.14
1394.35
1475.49
1490.33
2172.03
2953.03
2988.37
3099.85
3209.15
3407.51
     ZeroEnergy[kJ/mol]        -135.36
  ElectronicLevels[1/cm]                     1
   0.0000000000000000        2.0000000000000000
 End
 End
!************************************
Barrier L2  REACS IC
   RRHO
    Stoichiometry  C2N2H5
    Core PhaseSpaceTheory
      FragmentGeometry[angstrom]              7
  C                  0.05052800    0.70535200    0.00000000
 H                  0.59223700    1.06563400   -0.87850300
 H                 -0.94333700    1.18056700    0.00000000
 H                  0.59223700    1.06563400    0.87850300
 N                  0.05052800   -0.76080300    0.00000000
 H                 -0.44900100   -1.10916200    0.81210100
 H                 -0.44900100   -1.10916200   -0.81210100
      FragmentGeometry[angstrom]              2
 C                  0.00000000    0.00000000   -0.62766800
 N                  0.00000000    0.00000000    0.53800100
  SymmetryFactor     1.0000000000000000
          PotentialPrefactor[au]        94.4
          PotentialPowerExponent  6
      End
    Frequencies[1/cm]     16
316.27
851.39
```

```
979.67
1056.00
1170.62
1352.24
1461.12
1496.80
1518.88
1668.86
2152.08
2947.98
3051.48
3086.76
3488.87
3562.98
 ZeroEnergy[kJ/mol]            0.00
 ElectronicLevels[1/cm]                   1
  0.0000000000000000        2.0000000000000000
  End
Barrier L4  FC02 P3
   RRHO
    Stoichiometry  C2N2H5
    Core PhaseSpaceTheory
      FragmentGeometry[angstrom]               6
 C                 -0.62788900   -0.01188200    0.00000000
 H                 -0.97212900   -0.58181800   -0.87879400
 H                 -0.97212600   -0.58178500    0.87881800
 H                 -1.12710100    0.95935900   -0.00001700
 N                  0.80507200    0.15292000   -0.00000200
 H                  1.20318700   -0.79490500    0.00000700
      FragmentGeometry[angstrom]               3
 C                  0.00000000    0.00000000   -0.49814100
 H                  0.00000000    0.00000000   -1.56440500
 N                  0.00000000    0.00000000    0.65046400
  SymmetryFactor     1.0000000000000000
          PotentialPrefactor[au]        3703.38
          PotentialPowerExponent  6
      End
    Frequencies[1/cm]     16
256.20
785.56
785.56
949.26
1008.25
1042.84
1341.71
1394.87
1479.27
1486.82
2200.47
2939.89
2972.35
3082.63
3370.22
3457.47
 ZeroEnergy[kJ/mol]            -113.03
 ElectronicLevels[1/cm]                   1
```

```
  0.0000000000000000        2.0000000000000000
  End
 !************************************
Barrier TS3 IC FC02
  RRHO
   Geometry[angstrom]           9
 C                -1.38528600    0.15805100    0.14934700
 N                -2.42905700   -0.28650200   -0.10902500
 C                 1.76902700   -0.55603500   -0.00472200
 N                 1.09189200    0.72566900    0.03119700
 H                 1.28510700   -1.27251300   -0.67686700
 H                 2.79231900   -0.39866000   -0.35838500
 H                 1.79985800   -0.97739900    0.99933600
 H                 0.20713800    0.78922000    0.58673900
 H                 0.97328400    1.17308900   -0.87377400
  Core  RigidRotor
    SymmetryFactor   1.0
  End
  Tunneling    Eckart
    ImaginaryFrequency[1/cm]   299.22
    WellDepth[kJ/mol]          40.7
    WellDepth[kJ/mol]          176.1
    End
   Frequencies[1/cm]            20
75.03
102.52
143.38
211.48
300.00
689.54
961.21
1050.33
1099.16
1305.23
1441.69
1474.45
1501.09
1608.46
2198.72
2952.25
3018.71
3073.48
3133.36
3546.51
  ZeroEnergy[kJ/mol]  -28.43
    ElectronicLevels[1/cm]                      1
       0        2
 End
 !************************************
Barrier TS4 IC P4
  RRHO
   Geometry[angstrom]           9
 C                 1.54138300   -0.42609200    0.03378800
 N                 0.41103500    0.55615300   -0.03357600
 C                -0.84677200   -0.04590900   -0.21448800
 N                -1.95011000   -0.24089900    0.12901500
```

```
 H                  2.44972500    0.13919700    0.24123400
 H                  1.33736900   -1.10840100    0.85650700
 H                  1.62127700   -0.97638600   -0.90109900
 H                  0.62553400    1.35670000    1.01721700
 H                  0.57195000    1.21412100   -0.79772700
  Core  RigidRotor
    SymmetryFactor   1.0
  End
  Tunneling    Eckart
    ImaginaryFrequency[1/cm]  1231.85
    WellDepth[kJ/mol]         94.2
    WellDepth[kJ/mol]         88.2
    End
   Frequencies[1/cm]            20
171.48
202.59
389.15
543.78
750.62
804.85
903.73
1077.20
1102.87
1164.16
1228.20
1410.27
1440.53
1487.75
1504.63
2148.03
3064.99
3152.23
3165.33
3450.08
  ZeroEnergy[kJ/mol]  25.08
    ElectronicLevels[1/cm]                      1
       0        2
 End
  !*************************************
Barrier TS5 IC P5
  RRHO
   Geometry[angstrom]            9
 C                  1.76544900    0.23959900    0.00000000
 N                  0.00000000    0.76894900    0.00000000
 C                 -1.14942100   -0.14605200    0.00000000
 N                 -1.36655600   -1.30739400    0.00000000
 H                  2.35933700    1.14832400    0.00000000
 H                  1.84066500   -0.34872300    0.90647500
 H                  1.84066500   -0.34872300   -0.90647500
 H                 -0.08547300    1.37847400    0.81581400
 H                 -0.08547300    1.37847400   -0.81581400
  Core  RigidRotor
    SymmetryFactor   1.0
  End
  Tunneling    Eckart
    ImaginaryFrequency[1/cm]  746.35
```

```
    WellDepth[kJ/mol]         84.2
    WellDepth[kJ/mol]         153.4
    End
   Frequencies[1/cm]            20
83.72
147.85
236.40
508.19
574.54
635.53
845.04
873.70
1115.58
1164.31
1220.66
1436.81
1448.91
1594.80
2027.05
3088.27
3222.09
3234.43
3418.60
3499.22
  ZeroEnergy[kJ/mol]  15.07
    ElectronicLevels[1/cm]                      1
       0        2
 End
End
```
